# Network biomarkers in recovered psychosis patients who discontinued antipsychotics

**DOI:** 10.1038/s41380-023-02279-6

**Published:** 2023-09-29

**Authors:** Soyolsaikhan Odkhuu, Woo-Sung Kim, Uyanga Tsogt, Jie Shen, Sahar Cheraghi, Ling Li, Fatima Zahra Rami, Thi-Hung Le, Keon-Hak Lee, Nam-In Kang, Sung-Wan Kim, Young-Chul Chung

**Affiliations:** 1 Department of Psychiatry, Jeonbuk National University, Medical School, Jeonju, Korea; 2 Research Institute of Clinical Medicine of Jeonbuk National University-Biomedical Research Institute of Jeonbuk National University Hospital, Jeonju, Korea; 3 Department of Psychiatry, Jeonbuk National University Hospital, Jeonju, Korea; 4 Department of Psychiatry, Maeumsarang Hospital, Wanju, Korea; 5 Department of Psychiatry, Chonnam National University Medical School, Gwangju, Korea

**Keywords:** Diagnostic markers, Predictive markers, Schizophrenia

## Abstract

There are no studies investigating topological properties of resting-state fMRI (rs-fMRI) in patients who have recovered from psychosis and discontinued medication (hereafter, recovered patients [RP]). This study aimed to explore topological organization of the functional brain connectome in the RP using graph theory approach. We recruited 30 RP and 50 age and sex-matched healthy controls (HC). The RP were further divided into the subjects who were relapsed after discontinuation of antipsychotics (RP-R) and who maintained recovered state without relapse (RP-M). Using graph-based network analysis of rs-fMRI signals, global and local metrics and hub information were obtained. The robustness of the network was tested with random failure and targeted attack. As an ancillary analysis, Network-Based Statistic (NBS) was performed. Association of significant findings with psychopathology and cognitive functioning was also explored. The RP showed intact network properties in terms of global and local metrics. However, higher global functional connectivity strength and hyperconnectivity in the interconnected component were observed in the RP compared to HC. In the subgroup analysis, the RP-R were found to have lower global efficiency, longer characteristic path length and lower robustness whereas no such abnormalities were identified in the RP-M. Associations of the degree centrality of some hubs with cognitive functioning were identified in the RP-M. Even though network properties of the RP were intact, subgroup analysis revealed more altered topological organizations in the RP-R. The findings in the RP-R and RP-M may serve as network biomarkers for predicting relapse or maintained recovery after the discontinuation of antipsychotics.

## Introduction

Schizophrenia (SZ) and psychotic disorders are chronic and disabling mental illnesses. The life expectancy of patients with SZ is 15–20 years shorter than the life expectancy of the general population [1], and the age-standardized disability-adjusted life-years is reportedly 12.2% [2]. However, there is increasing evidence that psychotic patients, particularly individuals experiencing first-episode psychosis, can achieve favorable clinical outcomes, such as symptomatic remission and functional recovery [3, 4]. For individuals who achieved both symptomatic and functional recovery, there is a need to determine when and how antipsychotic medication should be discontinued. We recently published “Guidelines for discontinuation of antipsychotics in patients who recover from first-episode schizophrenia spectrum disorders” [5, 6]. In the guidelines, we suggested using stricter criteria when deciding whether to discontinue antipsychotics, rather than Andreasen’s criteria (which are most frequently used in discontinuation trials) [7]. Considering that most patients experience relapse after discontinuing antipsychotics [8, 9], another critical issue is relapse prediction. To tackle these issues, there is a need to analyze the clinical and biological characteristics of patients who meet strict criteria for full recovery and have successfully discontinued medication.

Multiple functional magnetic resonance imaging (fMRI) studies have examined neural correlates of clinical [10, 11] and symptomatic [12] recovery in psychotic and schizophrenic patients. They showed altered functional connectivity (FC) [11], as well as changes in the activities of specific brain regions, while the patients performed tasks [10, 12]. However, the participants in those studies did not meet our strict criteria for full recovery [3, 5]. SZ has been explained in terms of aberrant interactions among brain regions, leading to the degeneration of brain networks; this degeneration is referred to as disconnection syndrome [13–15]. Graph theory provides a powerful theoretical framework to test the dysconnectivity hypothesis in the context of SZ. Graph theory can characterize the topological properties of brain networks by analyzing whole-brain and local phenomena based on global network measurements. A meta-analysis of 13 fMRI studies showed that patients with SZ exhibited significant decreases in small-worldness (σ) and local organization [16]. However, findings regarding global efficiency (Eg) [17–20], nodal centrality [18, 21–23], and resilience [17, 24, 25] have been equivocal. Moreover, no studies have investigated brain network topological properties in patients who have recovered from psychosis and discontinued medication (hereafter, recovered patients [RP]) using resting-state functional magnetic resonance imaging (rs-fMRI). Based on the findings of previous graph analyses of functional brain networks in SZ patients, we hypothesized that there would be minimal or no differences in network topology and FC between RP (particularly individuals who did not experience any relapse, i.e., “recovered patients–maintained” [RP-M]) and healthy controls (HC). The investigation of functional brain networks in psychosis using graph theory may provide new insights regarding the network topology of RP. The present study compared both network topology and FC between RP and HC. Moreover, subgroup analysis was performed to identify network biomarkers for distinguishing among RP-M, patients who relapsed after discontinuation of antipsychotics (RP-R), and HC. The associations of aberrant connectivity with measures of psychopathology and cognitive function were also explored.

## Methods

### Participants

This study included 30 patients who had recovered from schizophrenia spectrum disorders or other psychotic disorders and attended the psychiatric outpatient clinic of Jeonbuk National University Hospital. These patients were participating in the Korea Early Psychosis Study (KEPS) [26] and were being regularly followed up (Supplementary Fig. S1). Diagnoses were made based on Diagnostic and Statistical Manual of Mental Disorders (DSM, Fifth Edition) criteria and the Structured Clinical Interview for DSM-IV [27, 28]. The criteria for full recovery were as follows: scores on Positive and Negative Syndrome Scale (PANSS) items P1, P2, P3, N1, N4, N6, G5, and G9 ≤ 2 [29, 30] and a total Social and Occupational Functioning Assessment Scale (SOFAS; [31]) score ≥71. Age- and sex-matched HC (n = 50) were recruited through advertisements. Patients were interviewed using the Structured Clinical Interview for DSM Disorders–Research Version [32]. All participants were required to have no previous or current psychiatric disorders, neurological disorders, or significant medical conditions, as well as no family history of psychotic disorders. Moreover, all participants were aged 19–60 years, were enrolled in the study on a voluntary basis, and had provided written informed consent. Handedness was assessed using the Edinburgh Handedness Inventory [33]. Depending on clinical status during follow-up (range: 1.5–109 months), participants were classified as “maintained” or” relapsed” (for relapse criteria, see [3]).

### Clinical assessment

Participants who met the criteria for full recovery were gradually tapered off their medications. After complete discontinuation of their medication, they were scanned and evaluated using the PANSS, Calgary Depression Scale for Schizophrenia [28, 34] SOFAS [31], and cognitive function tests (Supplementary Fig. S1). All participants were regularly followed up (usually at 2-month intervals) for 1–2 years. At each visit, they were provided with education regarding early signs and symptoms of relapse. Subsequently, participants were examined and evaluated on an ad hoc basis. To ensure interrater reliability (Cohen’s kappa ≥0.8), only psychiatrists with >2 years of experience participated in the study.

### Image acquisition and preprocessing

Structural magnetic resonance imaging and rs-fMRI scans were conducted at Jeonbuk National University Hospital using a 3 T Magnetom Verio scanner (Siemens, Erlangen, Germany). Three-dimensional T1-weighted images were obtained using a magnetization-prepared rapid gradient echo sequence (repetition time, 1900 ms; echo time, 2.5 ms; flip angle, 9°; field of view, 250 mm; image matrix: 256 × 246 mm; voxel size, 1.0 × 1.0 × 1.0 mm^3^; 176 slices). A 5-min resting-state scan consisting of 150 contiguous echo-planar imaging functional images (repetition time, 2000 ms; echo time, 30 ms; flip angle, 90°; field of view, 220 mm; image matrix, 64 × 64 mm; voxel size, 3.4 × 3.4 × 5.0 mm^3^; 26 slices) was also acquired. During the resting-state scan, participants were instructed to relax with their eyes closed, but not to sleep.

The rs-fMRI data were preprocessed using the GRETNA toolbox (https://www.nitrc.org/projects/gretna/) [35] and Statistical Parametric Mapping 12 software (https://www.fil.ion.ucl.ac.uk/spm/). The first 10 volumes were discarded to adjust for magnetization equilibrium. We implemented standardized procedures such as slice-timing, realignment, co-registration, spatial normalization, smoothing (using a 6-mm full-width at half-maximum isotropic Gaussian kernel), and linear detrending. Signals arising from cerebrospinal fluid, white matter, and the Friston-24 head motion parameters [36] were regressed out as covariates. A bandpass filter (0.01–0.08) was used to remove the effects of low- and high-frequency noise. The criterion for excessive head motion was a framewise displacement value > 0.5 mm. Participants were excluded from the analysis if >10% of their volumes showed excessive head motion [37]. For participants with excessive head motion in <10% of volumes, volume adjustment was performed by applying the nearest interpolation method to one of the first, and two of the last, volumes after scrubbing.

### Graph theory analysis

The FC matrix for each participant was constructed using the GRETNA 2.0 package [35] for MATLAB 2018a (MathWorks, Natick, MA, USA). For brain parcellation, we used the Dosenbach atlas, which covers 160 regions [38] and was generated based on a meta-analysis of task-related fMRI data. A 160 × 160 association matrix for each group was generated based on Pearson correlation coefficients. Absolute correlation matrices were thresholded at multiple densities to ensure an equal number of edges in all graphs (range: 0.06–0.37 [interval = 0.01]; for more details, see Table S1 in the Supplementary Materials) and converted into binary adjacency matrices. The following global network metrics were analyzed: small-worldness (σ), global efficiency (Eg), local efficiency (Eloc), clustering coefficient (Cp), normalized clustering coefficient (γ), characteristic path length (Lp), and normalized characteristic path length. Local metrics included degree centrality (Dc), betweenness centrality (Bc), and nodal efficiency. These metrics were estimated using the GRETNA 2.0 package. Definitions are presented in Supplementary Table S1. We calculated the areas under the curve (AUCs) for each participant and network metric at the sparsity threshold. Two-sample *t* test or analysis of variance (ANCOVA) comparisons were performed using the GRETNA package, with age and sex included as covariates. For global metrics, we performed a permutation test 10,000 times; this did not affect the results. Nodes exhibiting significant group differences were visualized by the BrainNet Viewer toolbox (https://www.nitrc.org/projects/bnv/).

Nodes were defined as hubs if the Dc was ≥ 1 standard deviation above the mean AUC of Dc for 160 nodes. For group comparisons, we calculated the mean AUC of Dc for all hubs. The numbers of hubs in the whole brain and subnetworks were determined using the AUC of the Dc matrix. Additionally, network resilience to targeted attack and random failure was evaluated. Targeted attack was assessed by removing nodes (in decreasing order of Dc), whereas random failure was assessed by randomly removing one node from the network (see Fig. S8 in the Supplementary Materials). Robustness was assessed according to the AUC of the giant connected component (GCC), relative to the number of nodes removed from each matrix. The threshold at which targeted attack or random failure had the greatest impact on GCC size (i.e., the minimum threshold) was used. Network disintegration was defined as a ≥ 1.5-fold decrease in GCC size.

### Functional brain network analysis

Global FC strength (mean of all pairwise correlation coefficients) was compared between groups. The network-based statistic (NBS) assumes that the brain is an integrated system, rather than a collection of individual components [39]; it also distinguishes subnetworks and interconnected components, allowing it to circumvent several issues that affect comparisons of networks based on thresholding procedures [19]. Moreover, by using information obtained via spatial clustering of abnormalities, the NBS also avoids the multiple comparison problem encountered when analyzing differences among many connections. The size of a significantly connected component (CC) can be controlled by cluster-based thresholding, which yields a t-value. In this study, we calculated the median cluster-defining threshold (t = 3.9; initial threshold = 3.6 ≤ t ≤ 4.2) using a published method [40]. In the subgroup analysis, the median F-value was 7.4 (initial threshold = 7.0 ≤ F ≤ 7.8). In post hoc tests, the t-values for the RP-M versus HC, RP-R versus HC, and RP-M versus RP-R group comparisons were 3.6 (3.0 ≤ t ≤ 4.2), 3.6 (3.0 ≤ t ≤ 4.2), and 2.5 (2.0 ≤ t ≤ 3.0), respectively. Group differences in the overall FC strength of CCs were analyzed using a *t* test or ANOVA, with age and sex included as covariates. The results were adjusted for multiple comparisons based on NBS correction [19]. We derived a mask matrix for each significant connection and applied it to the individual FC matrices in the MATLAB environment. Bonferroni correction was used for comparisons of multiple groups (*p* = 0.05/3). Additionally, we calculated the FC and nodal strength for significant CCs (see Supplementary Tables S13–S20).

### Statistical analysis

Demographic and clinical data were compared between RP (maintained and relapsed patients) and HC using a two-sample *t* test, ANOVA, or chi-squared test. The follow-up duration was the time between discontinuation of antipsychotics and the last outpatient visit or telephone evaluation. Three participants followed for <6 months were excluded from the relapse rate calculation. Only participants who were relapse-free during at least 2 years of follow-up were included in the subgroup analysis; thus, five participants were excluded from the subgroup analysis. Using SPSS software (ver. 24.0; IBM Corp, Armonk, NY, USA), we performed Spearman correlation analysis to evaluate the relationships of significant variables in the graph theory and functional brain network analyses with clinical variables (PANSS and cognitive function scores). *P* values < 0.05 were considered statistically significant, and Bonferroni correction was used for multiple comparisons.

## Results

### Demographic and clinical characteristics

There were no significant differences in age, sex, or education level between RP and HC (Table 1), or between RP-R and RP-M (Table 2). The recovered group had significantly lower composite scores for global cognitive function (*p* < 0.001), verbal memory (*p* < 0.001), and language (*p* < 0.001), compared with HC. In subgroup analysis, the same trends were observed during comparisons of maintained and relapsed groups with the HC (Table 2). It is of note that proportion of first episode were 84.6 and 75% in the RP-M and RP-R groups respectively.

**Table 1:** Demographic and clinical characteristics of the recovered patients with psychosis and healthy controls.

Characteristics	RP (*n* = 30)	HC (*n* = 50)	*p*-value
Age (years)	34.23 (10.09)	33.38 (10.01)	0.946^a^
Sex			
Male (%)	9 (30.00)	18 (36.00)	0.633^b^
Female (%)	21 (70.00)	32 (64.00)	
Education (years)	13.87 (2.42)	14.40 (1.68)	0.520^a^
Diagnoses			
SZ (%)	9 (30.00)	—	
Schizophreniform disorder (%)	11 (36.67)	—	
PNOS (%)	10 (33.33)	—	
PANSS			
Positive symptoms	7.17 (0.59)	—	
Negative symptoms	7.13 (0.43)	—	
General psychopathology	18.53 (3.54)	—	
Total	32.83 (4.10)	—	
CDSS	1.67 (2.32)	—	
SOFAS	78.00 (4.66)	—	
DI (months)	66.93 (49.52)	—	
Min	10.00		
Max	196.00		
Medication-free period before the scan (months)	8.77 (18.11)	—	
Min	0.20		
Max	69.53		
Time to relapse (*n* = 13)	12.15 (14.62)	—	
Min	2.00		
Max	58.50		
Duration of follow-up	51.20 (29.71)	—	
Min	1.50		
Max	109.00		
Relapse rate (%)		—	
1-year	9/27 (33.33)		
2-year	12/24 (50.00)		
3-year	9/21 (42.86)		

Data means ± standard deviation; ^a^Significant *T* statistic for the two-sample *t* test; ^b^Significant T statistic for the Chi-square test.

*CDSS* Calgary Depression Scale for Schizophrenia, *DI* Duration of Illness, *HC* Healthy Control, *PANSS* Positive and Negative Syndrome Scale, *PNOS* Psychotic disorder NOS, *RP* Recovered patients, *SZ* Schizophrenia, *SOFAS* Social and Occupational Functioning Assessment Scale.

**Table 2:** Demographic and clinical characteristics of the maintained and relapsed patients.

Characteristics	RP-M (*n* = 13)	RP-R (*n* = 12)	*p* value
Age (years)	29.69 (6.17)	36.58 (11.29)	0.068^a^
Sex			
Male (%)	6 (46.15)	1 (8.33)	0.104^b^
Female (%)	7 (53.85)	11 (91.67)	
Education (years)	14.69 (1.60)	13.92 (2.43)	0.352^a^
Handedness			
Left-handed (%)	0 (0.00)	1 (8.33)	0.731^b^
Right-handed (%)	12 (92.31)	10 (83.34)	
Ambidextrous (%)	1 (7.69)	1 (8.33)	
Diagnoses			
SCZ (%)	5 (38.46)	4 (33.33)	0.928^a^
Schizophreniform disorder (%)	4 (30.77)	3 (25.00)	
PNOS (%)	4 (30.77)	5 (41.67)	
First episode (%)	11 (84.6)	9 (75)	0.855^b^
PANSS			
Positive symptoms	7.00 (0.41)	7.42 (0.79)	0.108^a^
Negative symptoms	7.23 (0.60)	7.08 (0.29)	0.448^a^
General psychopathology	17.85 (2.73)	20.00 (4.47)	0.156^a^
Total	32.07 (3.28)	34.50 (5.14)	0.170^a^
CDSS	2.15 (2.48)	1.67 (2.53)	0.632^a^
SOFAS	78.85 (5.06)	78.33 (4.92)	0.800^a^
DI (months)	75.54 (56.94)	63.08 (46.92)	0.623^a^
Min	10.00	12.00	
Max	196.00	174.00	
Medication free period before the scan (months)	18.15 (24.93)	1.73 (1.66)	0.033^a^
Min	0.20	0.27	
Max	69.53	5.00	

Data means ± standard deviation; ^a^Significant *T* statistic for the two-sample *t* test; ^b^Significant T statistic for the Chi-square test.

*BCSS* Brief Core Schema Scale, *CDSS* Calgary Depression Scale for Schizophrenia, *DI* Duration of Illness, *PANSS* Positive and Negative Syndrome Scale, *PNOS* Psychotic disorder NOS, *RP-M* Recovered patients-maintained, *RP-R* Recovered patients-relapsed, *SCZ* Schizophrenia, *SOFAS* Social and Occupational Functioning Assessment Scale.

### Main group analysis

**Fig. 1:**
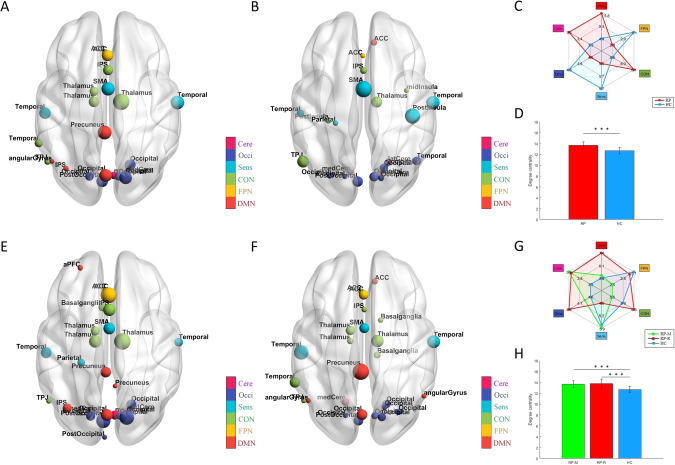
HUBs. **A** 22 hubs in the RP; (**B**) 23 hubs in HC; (**C**) Spider graph illustrating different numbers of hubs in each sub-network between two groups; (**D**) Comparison of Dc of all hubs between the RP (13.65 ± 0.64) and HC (12.66 ± 0.58), ^***^p < 0.0001; (**E**) 27 hubs in the RP-M; **(F**) 26 hubs in the RP-R; (**G**) Spider graph illustrating different numbers of hubs in each sub-network (calculated from the individual Dc matrix) among three subgroups; and (**H**) Comparison of Dc of all hubs among the RP-M (13.67 ± 0.64), RP-R (13.80 ± 0.84) and HC (12.66 ± 0.58), p < 0.0001 for the ANOVA (in the post-hoc tests, ^***^p < 0.0001 for the RP-M vs. HC and RP-R vs. HC). Note: The size and colors of the node indicate the magnitude of the degree centrality and subnetwork of the brain connectome; ACC anterior cingulate cortex, ANOVA analysis of variance, aPFC anterior prefrontal cortex, AUC area under the curve, Cere Cerebellum network, CON Cingulo-opercular network, Dc Degree centrality, DMN Default mode network, dFC dorsal frontal cortex, FPN Frontal parietal network, GCC giant connected component, HC Healthy controls, IPS intraparietal sulcus, mFC medial frontal cortex, Occi Occipital network, RP Recovered patients, RP-M Recovered patients with maintained, RP-R Recovered patients with relapsed, Sens Sensorimotor network, SMA supplementary motor area, TPJ Temporoparietal junction, vFC ventral frontal cortex.

**Fig. 2:**
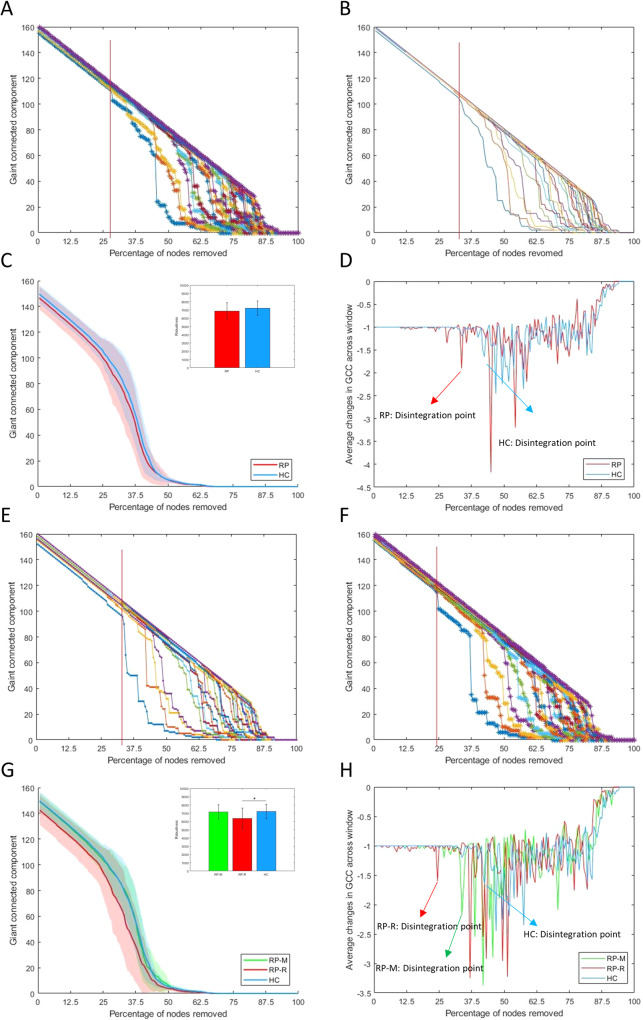
Robustness to targeted attack. In main group: Red line indicates the first disintegration point (**A**) in the RP and (**B**) in the HC at the threshold where the change of GCC size was most impactful (0.06); (**C**) Comparison of network robustness (AUC of GCC) between the RP (6863.2 ± 1037.4) and HC (7216.4 ± 869.2), *p* = 0.074; and (**D**) Arrows indicate disintegration point where average change in GCC was over 1.5 times the window prior. In subgroup: Red line indicates the first disintegration point (**E**) in the RP-M and (**F**) in the RP-R at the threshold where the change of GCC size was the most impactful (0.06); (**G**) Comparison of network robustness (AUC of GCC) among the RP-M (7168.9 ± 851.3), RP-R (6373.8 ± 1258.0) and HC (7216.4 ± 869.2), *p* = 0.0095 for the ANCOVA, (in the post-hoc tests, *p = 0.012 for the RP-R vs. HC); and (**H**) Arrows indicate disintegration point where the average change in GCC was over 1.5 times the window prior. Note: ANOVA Analysis of variance, AUC area under the curve, FC functional connectivity, GCC giant connected component, HC Healthy controls, RP Recovered patients, RP-M Recovered patients with maintained, RP-R Recovered patients with relapsed.

No significant group differences were observed in global measures (Supplementary Fig. S2). Regarding local measures, significant group differences were observed only for uncorrected data (Supplementary Tables S3–S5, Fig. S4). The total numbers of hubs were similar in RP and HC (*n* = 22 and 23, respectively; Fig. 1A, B, Supplementary Tables S9, S10). In RP, there were more hubs in the default mode network (DMN), cingulo-opercular network (CON), and cerebellum network (Cere), compared with HC (Fig. 1C). The mean Dc of hubs was significantly higher (*p* < 0.0001) in RP than in HC (Fig. 1D). In both RP and HC, the first point of network disintegration in response to a targeted attack corresponded to a threshold of 0.06. Network robustness did not significantly differ between the groups (*p* = 0.074) (Fig. 2C). However, the GCC began to exhibit instability when ~30% and ~40% of nodes were removed for RP and HC, respectively; with the removal of one node, the mean change in GCC size was approximately −1, but this change increased to approximately −2 and −1.7 for RP and HC, respectively, at the disintegration point (Fig. 2D). We did not observe a significant difference in random failure between RP and HC (Supplementary Fig. S8).

**Fig. 3:**
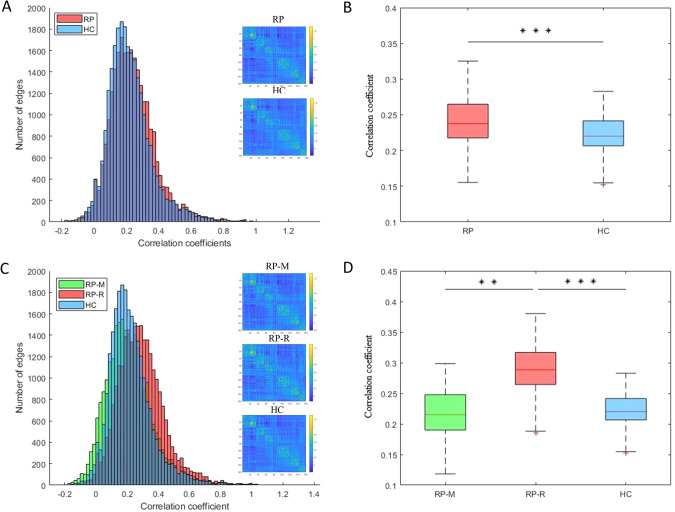
Global functional connectivity strength. **A** Distributions in the RP and HC; (**B**) Comparison between the RP (0.240 ± 0.035) and HC (0.223 ± 0.028), ^***^p < 0.0001; (**C**) Distributions in the RP-M, RP-R, and HC; and (**D**) Comparison among the RP-M (0.216 ± 0.039), RP-R (0.290 ± 0.040) and HC (0.223 ± 0.028), p < 0.0001 for the ANOVA (in the post-hoc tests, ^**^p = 0.0002 for the RP-M vs. RP-R and ^***^p < 0.0001 RP-R vs. HC). ANOVA analysis of variance, FC functional connectivity, HC Healthy controls, RP Recovered patients, RP-M Recovered patients with maintained, RP-R Recovered patients with relapsed.

**Fig. 4:**
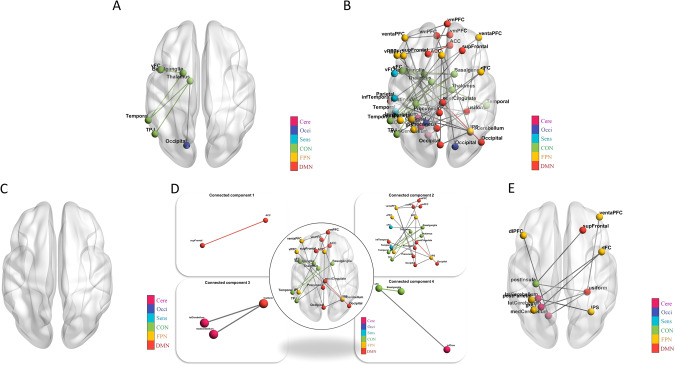
NBS analysis. **A** Altered connectomes between the RP and HC: at the median threshold (t = 3.9), the RP had significantly higher FC of a CC compared to HC (p = 0.031); (**B**) Altered connectomes among three subgroups: at the median threshold (F = 7.4), significantly different among the three subgroups (p = 0.043); and in the post-hoc tests, (**C**) No difference between the RP-M and HC; (**D**) the RP-R had significantly higher FC in the four CC compared to HC (p = 0.036, p < 0.001, p = 0.001, p = 0.001); and (**E**) the RP-R had significantly higher FC in a CC compared to the RP-M (p = 0.002). Note: The colors in the bar indicate the subnetwork of the brain connectome: ACC anterior cingulate cortex, ANCOVA Analysis of covariance, aPFC anterior prefrontal cortex, Cere Cerebellum network, CC connected component, CON Cingulo-opercular network, Dc Degree centrality, DMN Default mode network, dFC dorsal frontal cortex, FC functional connectivity, FPN Frontal parietal network, GCC giant connected component, HC Healthy controls, IPS intraparietal sulcus, mFC medial frontal cortex, NBS Network-Based Statistic, Occi Occipital network, RP Recovered patients, RP-M Recovered patients with maintained, RP-R Recovered patients with relapsed, Sens Sensorimotor network, SMA supplementary motor area, TPJ Temporoparietal junction, vFC ventral frontal cortex.

The global mean FC strength was significantly higher in RP than in HC (*p* < 0.0001; Fig. 3A, B). NBS analysis revealed significantly higher FC strength for CCs (five edges between six nodes) in RP than in HC (Fig. 4A). Cluster-defining thresholds were similar between the groups (Supplementary Tables S13, S14).

### Subgroup analysis

In post hoc tests, Eg and Lp were significantly different between RP-R and HC (both *p* = 0.01; Supplementary Fig. S3). Regarding local measures, only Bc of the left precuneus was significantly higher in RP-R than in HC at the false discovery rate-adjusted *p*-value (Supplementary Tables S6–S8; Supplementary Figs. S5–S7). The total numbers of hubs were 27, 26, and 23 in RP-M, RP-R, and HC, respectively (Fig. 1B, E, F Supplementary Tables S10–S12). In RP-M and RP-R, there were more hubs in DMN and CON, compared with HC (Fig. 1G). The mean Dc values of the hubs were significantly higher in RP-M and RP-R than in HC (*p* < 0.0001 and *p* < 0.0001, respectively; Fig. 1H). The minimum threshold for the three groups was similar to the threshold in the main analysis (i.e., 0.06; Fig. 2B, E, F). The AUC for network robustness to a targeted attack was significantly lower in RP-R than in HC (*p* = 0.011; Fig. 2G). The GCC began to exhibit instability when ~20%, ~35%, and ~40% of nodes were removed in RP-R, RP-M, and HC, respectively. With the removal of one node, the mean change in GCC was approximately −1, but this change increased to −1.7, −2.2, and −1.6 for RP-R, RP-M and HC, respectively, at the network disintegration point (Fig. 2H). We did not observe a significant difference in random failure among RP-M, RP-R, and HC (Fig. S8).

The global mean FC strength was significantly higher in RP-R than in RP-M or HC (*p* = 0.0002 and *p* < 0.0001, respectively; Fig. 3C, D). NBS analysis did not indicate a difference in FC between RP-M and HC (Fig. 4C). However, RP-R had significantly higher FC in four CCs compared with HC (CC1, *p* = 0.036 [1 edge between 2 nodes]; CC2, *p* < 0.001 [20 edges between 21 nodes]; CC3, *p* = 0.0012 [2 edges between 3 nodes]; CC4, *p* = 0.0012 [2 edges between 3 nodes]; Fig. 4D). RP-R had significantly higher FC in individual CCs, compared with RP-M (*p* = 0.0018 [11 edges between 12 nodes]; Fig. 4E). Cluster-defining thresholds were similar among the groups (Supplementary Tables S15–S20). When including medication free period as an additional covariate, the results were almost similar (Supplementary Table S25).

### Correlation analysis

**Fig. 5:**
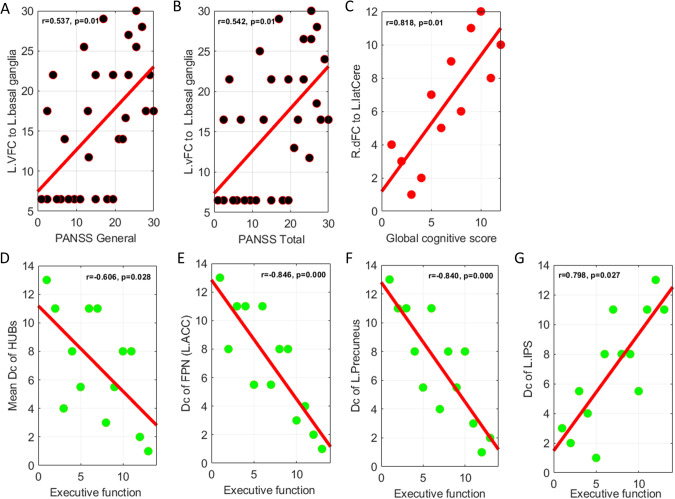
Correlation analysis. **A**, **B** FC strength of the L. vFC to L. basal ganglia was positively correlated with the general psychopathology and total scores of the PANSS in the RP; (**C**) FC strength of the R. dFC to L. latCere was positively correlated with the global cognition in the RP-R; and (**D**–**G**) in the RP-M, mean Dc of all hubs, Dc of the FPN and Dc of the L. precuneus were negatively correlated with the executive function. The Dc of the L. IPS was positively correlated with the executive function. Note: Dc degree centrality, FC functional connectivity, FPN Frontal parietal network, L.ACC Left anterior cingulate cortex, L.IPS Left intraparietal sulcus, L.latCere, Left lateral cerebellum, L.vFC Left ventral frontal cortex, p Bonferroni corrected *p* value, PANSS Positive and Negative Syndrome Scale Score, R.dFC Right dorsal frontal cortex, RP Recovered patients, RP-M Recovered patients-maintained, RP-R Recovered patients-relapsed.

In RP, FC strength between the left ventral frontal cortex and left basal ganglia was positively correlated with the general psychopathology and total scores of the PANSS (Bonferroni-corrected *p* = 0.010; Fig. 5A, B, Supplementary Table S23). Only the FC strength between the right dorsal frontal cortex and left lateral cerebellum was positively correlated with global cognition in RP-R (Bonferroni-corrected *p* = 0.011; Fig. 5C, Supplementary Table S24). In RP-M, the mean Dc values of all hubs (*p* = 0.028), the frontoparietal network (FPN [the FPN has only one hub, i.e., the left anterior cingulate cortex]; Bonferroni-corrected *p* < 0.0001), and the left precuneus (Bonferroni-corrected *p* < 0.0001) were negatively correlated with executive function. In contrast, Dc of the left intraparietal sulcus (Bonferroni-corrected *p* = 0.027) was positively correlated with executive function (Fig. 5D–G, Supplementary Table S22). However, we found no other significant correlations between clinical variables and the Dc values of the hubs (Supplementary Table S21) or other metrics of interest (data not shown).

## Discussion

Questions remain concerning whether patients with psychosis can fully recover and how to best predict recovery maintenance or relapse after the discontinuation of antipsychotics. To address these questions, we recruited participants who had met strict criteria for a full recovery and discontinued their medication. Using a graph theory approach, we observed significantly lower Eg and longer Lp in RP-R; higher mean Dc in hubs in RP, RP-M, and RP-R; and lower robustness in RP-R than in HC and RP-M. Moreover, RP-M and HC showed no significant differences in network robustness. The implications of these findings, and potential directions for future research, are discussed below.

The main group analysis revealed no significant differences in global metrics between RP and HC. The literature regarding Eg in SZ patients is conflicting, such that both increases [17, 18] and decreases [19, 20] are reported, although decreased σ [41] has consistently been observed. Considering the evidence for network abnormalities in SZ, the lack of altered global measures in our RP is encouraging and may indicate the normalization of network integration. However, these results must be confirmed in a larger study. Regarding node centrality, we observed no significant differences between RP and HC, although some nodes in RP showed altered centrality at uncorrected p-values. Notably, reduced node centrality was reported in a previous rs-fMRI study of SZ patients [18]; it was also reported in structural magnetic resonance imaging and diffusion tensor imaging studies [21–23]. Node centrality indicates how widely and rapidly a particular node affects others within the network. Our findings suggest that the networks of RP had similar efficiency (in terms of transferring information at the local level) to the networks of HC. However, whereas the hubs were concentrated in the CON, Occi, and DMN in RP, they were concentrated in the Occi, CON, and Sens in HC. A previous study showed that the hub distribution was more diffuse in the networks of SZ and HC [42]. In the present study, the hubs were mainly distributed in temporal and occipital regions, rather than the frontal region, in both RP and HC. Two reviews also identified few hubs in the frontal cortex; the findings in those reviews also suggested that the proliferation of hubs in other regions is a typical feature of the networks of SZ patients [41, 43]. Notably, we found that the mean Dc of all hubs was significantly higher in RP than in HC. Previously, decreased centrality of hubs in frontal, temporal, and limbic regions was reported [44]. Considering the key role of hubs in information integration [45, 46], hidden or subtle brain network dysfunction at the hub level may occur in RP, despite the absence of abnormalities in global and local measures. This postulation is supported by our finding that the Dc values of some hubs were significantly associated with PANSS and cognitive test scores, although the associations disappeared after p value correction. Importantly, although RP and HC did not differ in terms of network robustness, network disintegration in RP occurred at an earlier stage after fewer attacks, suggesting lower resilience. Lower network resilience was also reported in first-episode antipsychotic-naïve psychosis patients [17, 47]. In the present study, functional brain network analysis revealed higher global and subnetwork FC strength in RP than in HC. Moreover, the strength of each node in the subnetworks was greater in RP than in HC. Hyperconnectivity of specific brain regions is presumed to reflect increased functional integration, but it may also indicate excessive salience of, and/or heightened focus on, internal stimuli [48]. Globally enhanced FC was observed after administration of the N-methyl-D-aspartate (NMDA) receptor antagonist ketamine to healthy volunteers [49]. Our findings suggest that hyperconnectivity increases the risk of relapse in RP; evidence for this increased risk was provided by the correlations of ventral frontal cortex–basal ganglia connectivity with the total and general psychopathology PANSS scores.

In our subgroup analysis, lower Eg and longer Lp were observed in RP-R than in HC, which suggests brain network inefficiency in RP who relapsed after the scan (i.e., RP-R). Regarding local measures, only Bc of the left precuneus was significantly different between RP-R and HC. According to the uncorrected results, more nodes were significantly different between RP-R and HC than between RP-M and HC. The precuneus is involved in a wide range of highly integrated tasks, including tasks involving visuospatial imagery, episodic memory retrieval, and self-related mental representations [50]. Thus, higher Bc in the precuneus may indicate excessive information transfer when processing self-related mental representations in the RP-R. In summary, our findings suggest that lower Eg, longer Lp, and higher Bc of the precuneus could serve as biomarkers of relapse after the discontinuation of antipsychotics. The hubs were mainly distributed in the CON and DMN in both RP-R and RP-M, but this distribution was not observed in HC. Moreover, the mean Dc of all hubs was significantly higher in RP-R and RP-M than in HC. Along with the FPN, the CON is an important executive network [51]. The DMN plays key roles in various cognitive processes, including self-referential processing [52]. Hubs have high metabolic demands and may be susceptible to metabolic disease processes, such as oxidative stress [53, 54]. Our findings imply that hubs in the CON and DMN are subjected to severe stress in RP-R and RP-M. Surprisingly, the mean Dc of all hubs, and the Dc values of several individual hubs in the FPN, CON, and DMN, were associated with cognitive function only in RP-M. In a recent study, high Dc values for nodes in the DMN and CON were strongly associated with worse working memory performance [55]. Importantly, we found that the AUC for GCC size and the network disintegration point was significantly smaller in RP-R than in HC. If replicated, this finding could aid the prediction and prevention of relapse after the discontinuation of antipsychotics. The results for RP-M also have important clinical implications regarding the decision to discontinue antipsychotic medication, as well as the subsequent follow-up duration. The functional brain network analysis revealed higher global and subnetwork FC strength in RP-R than in HC or RP-M. Moreover, nodal strength within individual subnetworks was typically stronger in RP than in HC or RP-M. Furthermore, the significant associations (at uncorrected p-values) of some connections with positive symptoms and global cognitive scores suggest that altered connectivity in RP-R could serve as a neural marker for relapse. Notably, there were no significant differences between RP-M and HC. Taken together, these results suggest that the NBS can be used to differentiate RP-R from RP-M and HC.

Several limitations of this study should be noted. First, the sample size was small, particularly for the subgroup analysis; this can be attributed to the difficulty of recruiting participants who have recovered from psychosis and are no longer taking medication. Second, the proportion of patients with multiple episodes was slightly higher in the RP-R (25%) compared to the RP-M (15.4%) albeit no statistical difference. This issue warrants further investigation with a larger sample size. Third, the diagnoses were heterogeneous and patients with a psychotic disorder not otherwise specified (PNOS) were included. However, all such patients (patients with delusions, n = 9; patients with auditory hallucinations, n = 1) had a stable diagnosis over a 2-year period. Fourth, the variation in PANSS scores among RP was small, which may have biased the results of the correlation analyses [56]. Fifth, in the NBS analysis, we used raw rather than absolute values, considering assumptions that have been made regarding whether negative correlations should be included in analyses of fMRI data [57]. However, additional analyses using absolute values did not yield different results, except for a higher number of connections. Despite these caveats, this was the first graph-based network analysis of rs-fMRI data focusing on patients with psychosis who met strict criteria for full recovery and had discontinued their medication. The clinical application of our findings might be that when network biomarkers for relapse are identified after the discontinuation, more thorough psychoeducation for relapse signs and more frequent follow up should be provided.

In conclusion, RP showed intact global and local network properties, although hyperconnectivity was observed in interconnected components, compared with HC. However, in the subgroup analysis, RP-R had lower Eg, longer Lp, and less robust networks than RP-M. These findings may aid the prediction of relapse after the discontinuation of antipsychotics.

### Supplementary information

Supplemental tables and figure legends

Suppl Figure S1

Suppl Figure S2

Suppl Figure S3

Suppl Figure S4

Suppl Figure S5

Suppl Figure S6

Suppl Figure S7

Suppl Figure S8
