# Supplementary material for: Network biomarkers in recovered psychosis patients who discontinued antipsychotics
Source: Mol Psychiatry. 2023 Sep 29;28(9):3717–26. doi: 10.1038/s41380-023-02279-6 (PMC10730417; doi:10.1038/s41380-023-02279-6)
Supplement: Supplementary file 1 — Supplemental tables and figure legends [file 41380_2023_2279_MOESM1_ESM.doc]

**Supplemental tables**

| **Table S1. Measurements of the graph theory** | | | |
| --- | --- | --- | --- |
| **Measurements** | **Character** | **Meaning** | **Formula** |
| Number of nodes | N | 160 regions. | Dosenbach's atlas |
| Adjacency matrix | 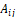 | Pearson correlation matrix. | 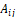 |
| Global efficiency | 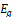 | Characterization of connectome integration. It means measuring the transfer of parallel information at a relatively low cost. | 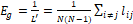; 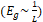; |
| Local efficiency | 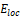 | How well neighbours of a node communicate with each other node is removed. | 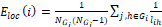; 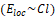; |
| Clustering coefficient | 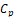 | A measure of local connectivity. | 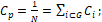 |
| Normalized clustering coefficient | 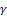 | Relative to the random graph (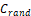). | 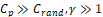, where 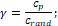 |
| Characteristic path length |  | The average shortest path length between all possible pairs of nodes in a network. | 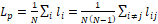; |
| Normalized characteristic path length | 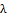 | Relative to the random graph (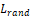). | 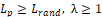, where 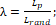 |
| Small-worldness | 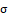 | Evaluating the balance of segregation and integration. | 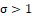, where 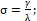 |
| Degree centrality/degree | Dc / 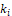 | The number of connections for each node. | 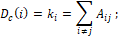 |
| Betweenness centrality | Bc | The fraction of the shortest paths that pass-through a given node or edge. It affects that one node has over the flow of information over all other nodes in the network. | 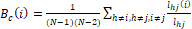; |
| Nodal efficiency | Ne | The ability of a node to propagate information with other nodes not only their neighbours in a network. Higher nodal efficiency is indicative of higher integration in the brain. | 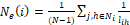; |
| Hub |  | Node with the highest degree. | 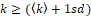; |
| Robustness |  | The robustness parameter is defined as the AUC of the giant connected component (GCC) versus the number of nodes removed curve. |  |
| The area under the curve | AUC | The integrated AUC metric is sensitive for detecting topological alterations of brain functional connectomes. | 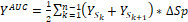; |
| Mean degree centrality | 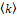 | Average of all the node degrees. | 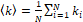; |
| Minimum mean degree centrality | 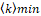 | Mean degree when the highest sparse network without fragmentation. | 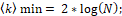 |
| Number of existing edges | 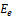 | When the network is sparsely connected. | 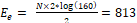; |
| Number of maximum edges | 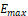 | When the network is fully connected. | 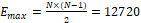; |
| Minimum sparsity threshold | Sp (min) | When the highest sparse network is without fragmentation. | S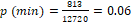; |
| Maximum sparsity threshold | Sp (max) | Small-worldness is larger than 1.1 for each subject at each threshold. | Sp (max) = 0.37; |
| **Note:** Only global metrics were normalized by the metric of the random graph; 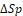 , step of sparsity threshold; Sparsity range was defined [0.06-0.37, 0.01]; Y, graph theory metrics; | | | |

| **Table S2. Cognitive functioning in the recovered, maintained, and relapsed patients** | | | | | | | | | | | | |
| --- | --- | --- | --- | --- | --- | --- | --- | --- | --- | --- | --- | --- |
|  | RP  (n = 30) |  | RP-M  (n = 13) |  | RP-R  (n = 12) |  | HC  (n = 50) | p-value^a^ | p-value^b^ | 2 vs 4 | 3 vs 4 | 2 vs 3 |
| Global cognitive function | -0.53(0.53) |  | -0.61(0.51) |  | -0.48(0.56) |  | 0.00(0.44) | <0.001 | <0.001 | <0.001 | 0.007 | 1.000 |
| Attention |  |  |  |  |  |  |  |  |  |  |  |  |
| Stroop test |  |  |  |  |  |  |  |  |  |  |  |  |
| Color-word total time | 16.48(4.64) |  | 17.08(4.45) |  | 15.19(3.69) |  | 16.71(7.10) | 0.874 | 0.712 | 1.000 | 1.000 | 1.000 |
| Color of color-word total time | 25.48(10.23) |  | 24.89(6.37) |  | 27.59(14.40) |  | 24.07(9.62) | 0.538 | 0.556 | 1.000 | 0.842 | 1.000 |
| Composite score | -0.06(0.74) |  | -0.07(0.56) |  | -0.08(0.94) |  | 0.00(0.83) | 0.751 | 0.936 | 1.000 | 1.000 | 1.000 |
| Auditory CPT |  |  |  |  |  |  |  |  |  |  |  |  |
| Correct response | 127.30(10.32) |  | 125.23(11.12) |  | 128.08(11.15) |  | 131.58(4.10) | 0.011 | 0.014 | 0.017 | 0.397 | 0.965 |
| Commission error | 4.50(3.94) |  | 6.00(4.51) |  | 3.25(2.70) |  | 4.94(12.32) | 0.850 | 0.800 | 1.000 | 1.000 | 1.000 |
| Composite score | -0.50(1.35) |  | -0.82(1.48) |  | -0.36(1.40) |  | 0.00(0.83) | 0.072 | 0.046 | 0.048 | 0.895 | 0.852 |
| Composite score | 0.28(0.81) |  | -0.44(0.86) |  | -0.22(0.87) |  | 0.00(0.57) | 0.102 | 0.100 | 0.117 | 0.967 | 1.000 |
| Verbal memory |  |  |  |  |  |  |  |  |  |  |  |  |
| Total recall | 51.93(10.67) |  | 49.62(11.91) |  | 52.00(9.45) |  | 93.12(12.72) | <0.001 | <0.001 | <0.001 | <0.001 | 1.000 |
| Learning slope | 4.93(2.12) |  | 4.15(1.91) |  | 5.67(2.01) |  | 6.24(2.08) | 0.008 | 0.006 | 0.005 | 1.000 | 0.204 |
| Delayed recall | 10.73(3.00) |  | 10.85(3.18) |  | 10.67(2.84) |  | 10.86(2.22) | 0.842 | 0.971 | 1.000 | 1.000 | 1.000 |
| Composite score | -1.31(0.90) |  | -1.48(0.96) |  | -1.20(0.88) |  | 0.00(0.79) | <0.001 | <0.001 | <0.001 | <0.001 | 1.000 |
| Executive function |  |  |  |  |  |  |  |  |  |  |  |  |
| Categories completed | 5.67(0.80) |  | 5.92(0.28) |  | 5.42(1.08) |  | 5.40(1.43) | 0.289 | 0.403 | 0.557 | 1.000 | 0.953 |
| Perseverative error | 9.60(6.39) |  | 7.85(2.41) |  | 10.50(6.92) |  | 12.18(10.83) | 0.184 | 0.327 | 0.428 | 1.000 | 1.000 |
| Composite score | 0.21(0.54) |  | 0.38(0.16) |  | 0.08(0.65) |  | 0.00(0.88) | 0.235 | 0.285 | 0.342 | 1.000 | 1.000 |
| Language |  |  |  |  |  |  |  |  |  |  |  |  |
| Animals | 16.60(4.45) |  | 15.85(5.13) |  | 16.83(3.83) |  | 19.92(4.31) | 0.001 | 0.005 | 0.012 | 0.096 | 1.000 |
| Stationery | 16.27(4.76) |  | 14.38(4.72) |  | 17.92(4.44) |  | 22.06(4.92) | <0.001 | <0.001 | <0.001 | 0.028 | 0.213 |
| “ㄱ” | 11.33(3.64) |  | 11.38(3.73) |  | 11.42(3.75) |  | 13.82(4.86) | 0.011 | 0.100 | 0.266 | 0.310 | 1.000 |
| “ㅅ” | 10.60(3.72) |  | 10.31(4.52) |  | 10.92(2.84) |  | 12.66(4.22) | 0.032 | 0.126 | 0.216 | 0.583 | 1.000 |
| “ㅇ” | 8.80(3.11) |  | 8.00(3.39) |  | 9.67(2.50) |  | 12.06(4.22) | <0.001 | 0.003 | 0.004 | 0.175 | 0.858 |
| Composite score | -0.74(0.61) |  | -0.90(0.67) |  | -0.61(0.51) |  | 0.00(0.64) | <0.001 | <0.001 | <0.001 | 0.011 | 0.710 |
| Data are mean (± standard deviation); RP, RP-M, RP-R, and HC are labeled as 1, 2, 3, and 4, respectively; ^a^Two-sample t-test between 1 and 4; ^b^ANOVA among 2, 3, and 4. | | | | | | | | | | | | |

| **Table S3. Degree centrality between the RP (n=30) and HC (n=50).** | | | | | | | |  |
| --- | --- | --- | --- | --- | --- | --- | --- | --- |
| **Index** | **ROIs** | **Coordinates** | | | **HC** | **RP** | **p-unc** |  |
| **RP > HC** | | | | | | | |  |
| 14 | Right posterior cingulate | 0.6 | -26.4 | 30.6 | 8.23(3.69) | 11.33(4.72) | 0.0013 |  |
| 16 | Left precuneus | -3 | -38.25 | 45 | 12.03(3.56) | 14.46(4.72) | 0.0058 |  |
| 33 | Left occipital | -2.0625 | -75 | 32.0625 | 11.25(4.21) | 14.22(4.90) | 0.0042 |  |
| 70 | Left thalamus | -12 | -3 | 12.75 | 9.75(4.52) | 13.45(4.65) | 0.0007 |  |
| 156 | Right medial cerebellum | 5.0625 | -75 | -11.0625 | 9.97(4.69) | 13.06(5.08) | 0.0095 |  |
| **RP < HC** | | | | | | | |  |
| 62 | Left anterior insula | -36 | 18 | 2.25 | 11.91(4.35) | 9.27(4.28) | 0.0059 |  |
| 69 | Right mid-insula | 36.9375 | -2.0625 | -3 | 12.17(4.01) | 9.89(5.15) | 0.0193 |  |
| 83 | Left parietal | -54.9375 | -44.0625 | 30 | 11.81(5.14) | 9.56(4.18) | 0.0494 |  |
| 96 | Left mid-insula | -42 | -3 | 11.25 | 10.99(4.19) | 8.48(4.16) | 0.0068 |  |
| 113 | Right posterior insula | 42 | -24 | 17.25 | 13.90(5.50) | 11.08(5.18) | 0.0222 |  |
| 138 | Left posterior occipital | -36.6 | -83.4 | -2.4 | 10.93(3.89) | 9.25(4.10) | 0.0470 |  |
| 139 | Left posterior occipital | -29.4 | -87.6 | 8.4 | 11.51(3.82) | 9.25(3.88) | 0.0197 |  |
| 145 | Left inferior cerebellum | -36.9375 | -54 | -36.9375 | 10.04(5.16) | 7.36(4.61) | 0.0106 |  |
| Comparison of degree centrality (AUC of Dc) between the RP and HC using a two-sample t-test (p<0.05) with covariates (age and sex). Significant results were found only at the uncorrected level.  **Note:** AUC, area under the curve; Data are mean (± standard deviation); Dc, degree centrality; HC, Healthy controls; RP, Recovered patients; | | | | | | | |  |
|  |

| **Table S4. Betweenness centrality between the RP (n=30) and HC (n=50).** | | | | | | | |  |
| --- | --- | --- | --- | --- | --- | --- | --- | --- |
| **Index** | **ROIs** | **Coordinates** | | | **HC** | **RP** | **p-unc** |  |
| **RP > HC** | | | | | | | |  |
| 14 | Right posterior cingulate | 0.6 | -26.4 | 30.6 | 16.23(19.57) | 27.95(22.43) | 0.0140 |  |
| 16 | Left precuneus | -3 | -38.25 | 45 | 34.26(19.60) | 47.53(31.88) | 0.0133 |  |
| 33 | Left occipital | -2.0625 | -75 | 32.0625 | 30.32(18.56) | 43.05(24.67) | 0.0092 |  |
| 70 | Left thalamus | -12 | -3 | 12.75 | 22.76(15.40) | 35.92(26.72) | 0.0057 |  |
| 99 | Right precentral gyrus | 45.9375 | -8.0625 | 24 | 8.83(8.51) | 13.38(10.83) | 0.0377 |  |
| 157 | Right medial cerebellum | 14.25 | -75 | -21 | 34.52(23.25) | 50.99(38.98) | 0.0222 |  |
| 159 | Left inferior cerebellum | -6 | -78.75 | -33 | 25.70(16.73) | 39.21(29.66) | 0.0131 |  |
| **RP < HC** | | | | | | | |  |
| 11 | Left superior frontal cortex | -15.9375 | 29.0625 | 54 | 23.73(17.21) | 15.97(12.84) | 0.0398 |  |
| 62 | Left anterior insula | -36 | 18 | 2.25 | 27.67(17.23) | 18.48(14.66) | 0.0117 |  |
| 82 | Right temporal | 42.6 | -42.6 | 8.4 | 15.14(16.01) | 8.70(10.10) | 0.0340 |  |
| 96 | Left mid-insula | -42 | -3 | 11.25 | 25.01(20.25) | 14.94(13.73) | 0.0139 |  |
| 124 | Right temporal | 45.6 | -62.4 | 5.4 | 33.90(21.99) | 24.24(16.44) | 0.0411 |  |
| 125 | Left occipital | -44.0625 | -63 | -6.9375 | 27.29(21.37) | 18.18(9.74) | 0.0291 |  |
| 145 | Left inferior cerebellum | -36.9375 | -54 | -36.9375 | 25.71(28.24) | 13.90(13.05) | 0.0311 |  |
| Comparison of betweenness centrality (AUC of Bc) between the RP and HC using a two-sample t-test (p<0.05) with covariates (age and sex). Significant results were found only at the uncorrected level.  **Note:** AUC, area under the curve; Bc, betweenness centrtality; Data are mean (± standard deviation); HC, Healthy controls; RP, Recovered patients; | | | | | | | |  |
|  |

| **Table S5. Nodal efficiency between the RP (n=30) and HC (n=50).** | | | | | | | |  |
| --- | --- | --- | --- | --- | --- | --- | --- | --- |
| **Index** | **ROIs** | **Coordinates** | | | **HC** | **RP** | **p-unc** |  |
| **RP > HC** | | | | | | | |  |
| 14 | Right posterior cingulate | 0.6 | -26.4 | 30.6 | 0.164(0.020) | 0.176(0.027) | 0.0272 |  |
| 16 | Left precuneus | -3 | -38.25 | 45 | 0.182(0.015) | 0.191(0.017) | 0.0118 |  |
| 33 | Left occipital | -2.0625 | -75 | 32.0625 | 0.179(0.17) | 0.190(0.019) | 0.0098 |  |
| 70 | Left thalamus | -12 | -3 | 12.75 | 0.170(0.025) | 0.187(0.019) | 0.0023 |  |
| 156 | Right medial cerebellum | 5.0625 | -75 | -11.0625 | 0.172(0.023) | 0.185(0.020) | 0.0096 |  |
| **RP > HC** | | | | | | | |  |
| 59 | Right ventral frontal cortex | 51 | 23.0625 | 8.0625 | 0.177(0.028) | 0.167(0.025) | 0.0331 |  |
| 62 | Left anterior insula | -36 | 18 | 2.25 | 0.181(0.020) | 0.168(0.023) | 0.0080 |  |
| 69 | Right mid-insula | 36.9375 | -2.0625 | -3 | 0.183(0.026) | 0.169(0.029) | 0.0095 |  |
| 83 | Left parietal | -54.9375 | -44.0625 | 30 | 0.180(0.023) | 0.170(0.020) | 0.0498 |  |
| 96 | Left mid-insula | -42 | -3 | 11.25 | 0.177(0.019) | 0.160(0.037) | 0.0072 |  |
| 113 | Right posterior insula | 42 | -24 | 17.25 | 0.189(0.021) | 0.177(0.022) | 0.0178 |  |
| 118 | Left temporal | -41.4 | -36.6 | 15.6 | 0.176(0.027) | 0.163(0.028) | 0.0113 |  |
| 138 | Left posterior occipital | -36.6 | -83.4 | -2.4 | 0.177(0.026) | 0.166(0.027) | 0.0237 |  |
| 139 | Left posterior occipital | -29.4 | -87.6 | 8.4 | 0.179(0.027) | 0.170(0.017) | 0.0225 |  |
| 145 | Left inferior cerebellum | -36.9375 | -54 | -36.9375 | 0.170(0.032) | 0.154(0.035) | 0.0371 |  |
| Comparison of nodal efficiency (AUC of Ne) between the RP and HC using a two-sample t-test (p<0.05) with covariates (age and sex). Significant results were found at the uncorrected level.  **Note:** AUC, area under the curve; Data are mean (± standard deviation); HC, Healthy controls; Ne, nodal efficiency; RP, Recovered patients; | | | | | | | |  |
|  |

| **Table S6. Degree centrality among the RP-M (n=13), RP-R (n=12), and HC (n=50).** | | | | | | | | | | | | | |  |
| --- | --- | --- | --- | --- | --- | --- | --- | --- | --- | --- | --- | --- | --- | --- |
| **Index** | **ROIs** | **Coordinates** | | | **RP-M** | **RP-R** | **HC** | **1 vs 2 vs 3** | | **1 vs 3** | **2 vs 3** | **1 vs 2** |  | |
|  |  |  |  |  |  |  |  | **p-unc** | **p-FDR** | **p-unc** | **p-unc** | **p-unc** |  | |
| 14 | Right posterior cingulate | 0.6 | -26.4 | 30.6 | 10.025(3.665) | 12.372(6.025) | 8.228(3.690) | 0.0110 | 0.4634 | 0.1750 | 0.0281 | 0.5636 |  | |
| 16 | Left precuneus | -3 | -38.25 | 45 | 13.781(3.558) | 15.990(5.322) | 12.028(3.558) | 0.0051 | 0.4060 | 0.2490 | 0.0107 | 0.6564 |  | |
| 33 | Left occipital | -2.0625 | -75 | 32.0625 | 14.029(4.093) | 14.498(5.871) | 11.247(4.207) | 0.0216 | 0.4945 | 0.0697 | 0.1359 | 1.0000 |  | |
| 50 | Left posterior parietal | -35.0625 | -45.9375 | 48 | 7.460(3.386) | 11.191(5.012) | 11.043(3.586) | 0.0255 | 0.4946 | 0.0116 | 1.0000 | 0.7634 |  | |
| 57 | Right ventral prefrontal cortex | 33.6 | 32.4 | 6.6 | 5.922(4.061) | 4.803(3.382) | 7.503(3.95) | 0.0347 | 0.5548 | 1.0000 | 0.0674 | 0.1950 |  | |
| 62 | Left anterior insula | -36 | 18 | 2.25 | 8.874(2.993) | 9.014(5.394) | 11.914(4.352) | 0.0117 | 0.4634 | 1.0000 | 1.0000 | 1.0000 |  | |
| 69 | Right mid-insula | 36.9375 | -2.0625 | -3 | 11.591(5.211) | 7.491(4.435) | 12.170(4.013) | 0.0145 | 0.4634 | 1.0000 | 0.0072 | 0.9123 |  | |
| 70 | Left thalamus | -12 | -3 | 12.75 | 13.516(3.413) | 14.078(5.977) | 9.745(4.516) | 0.0028 | 0.4060 | 0.0044 | 0.0730 | 1.0000 |  | |
| 96 | Left ventral frontal cortex | -42 | -3 | 11.25 | 9.697(4.615) | 7.193(3.781) | 10.996(4.192) | 0.0194 | 0.4945 | 0.7862 | 0.0120 | 1.0000 |  | |
| 145 | Left inferior cerebellum | -36.9375 | -54 | -36.9375 | 8.037(4.696) | 6.380(4.327) | 10.035(5.157) | 0.0278 | 0.4946 | 0.4832 | 0.0465 | 1.0000 |  | |
| RP-M, RP-R, and HC are labeled as 1, 2, and 3, respectively; Comparison of degree centrality (AUC of Dc) among the 1, 2, and 3, ANCOVA (p<0.05) with covariates (age and sex). Significant results were found only at the uncorrected level.  **Note:** ANCOVA, Analysis of covariance; AUC, area under the curve; Data are mean (± standard deviation); Dc, degree centrality; False discovery rate; RP-M, Recovered patients with maintained; RP-R, Recovered patients with relapsed; HC, Healthy controls; | | | | | | | | | | | | | |  |
|  |  |  |  |  |  |  |  |  |  |  |  |  |  |  |

| **Table S7. Betweenness centrality among the RP-M (n=13), RP-R (n=12), and HC (n=50).** | | | | | | | | | | | | | |  |
| --- | --- | --- | --- | --- | --- | --- | --- | --- | --- | --- | --- | --- | --- | --- |
| **Index** | **ROIs** | **Coordinates** | | | **RP-M** | **RP-R** | **HC** | **1 vs 2 vs 3** | | **1 vs 3** | **2 vs 3** | **1 vs 2** |  | |
|  |  |  |  |  |  |  |  | **p-unc** | **p-FDR** | **p-unc** | **p-unc** | **p-unc** |  | |
| 14 | Right posterior cingulate | 0.6 | -26.4 | 30.6 | 22.120(18.210) | 35.745(28.724) | 16.234(19.573) | 0.0250 | 0.4992 | 0.6995 | 0.0497 | 0.2855 |  | |
| 16 | Left precuneus | -3 | -38.25 | 45 | 36.095(22.559) | 65.829(38.063) | 34.256(19.599) | 0.0001 | 0.0192 | 1.0000 | 0.0002 | 0.1013 |  | |
| 33 | Left occipital | -2.0625 | -75 | 32.0625 | 39.017(21.499) | 51.767(29.177) | 30.316(18.563) | 0.0051 | 0.2731 | 0.4126 | 0.0058 | 0.5991 |  | |
| 62 | Left anterior insula | -36 | 18 | 2.25 | 13.474(8.993) | 21.507(14.805) | 27.669(17.227) | 0.0047 | 0.2731 | 0.0091 | 0.7446 | 0.0191 |  | |
| 70 | Left thalamus | -12 | -3 | 12.75 | 37.800(24.460) | 36.840(32.682) | 22.762(15.404) | 0.0149 | 0.4360 | 0.0040 | 0.2224 | 1.0000 |  | |
| 96 | Left mid-insula | -42 | -3 | 11.25 | 21.045(17.281) | 8.668(6.009) | 25.007(20.246) | 0.0158 | 0.4360 | 1.0000 | 0.0095 | 0.2159 |  | |
| 155 | Right inferior cerebellum | 33 | -72.75 | -30 | 21.962(14.600) | 41.021(40.251) | 24.173(17.150) | 0.0216 | 0.4939 | 1.0000 | 0.0600 | 0.1579 |  | |
| 159 | Left inferior cerebellum | -6 | -78.75 | -33 | 45.901(31.951) | 30.102(13.791) | 25.704(16.730) | 0.0164 | 0.4360 | 0.0223 | 1.0000 | 1.0000 |  | |
| RP-M, RP-R, and HC are labeled as 1, 2, and 3, respectively; Comparison of betweenness centrality (AUC of Bc) among the 1, 2, and 3, ANCOVA (p<0.05) with covariates (age and sex). Significant results were found at the uncorrected level except the left precuneus.  **Note:** ANCOVA, Analysis of covariance; AUC, area under the curve; Bc, betweenness centrality; Data are mean (± standard deviation); False discovery rate; HC, Healthy controls; RP-M, Recovered patients with maintained; RP-R, Recovered patients with relapsed; | | | | | | | | | | | | | |  |
|  |  |  |  |  |  |  |  |  |  |  |  |  |  |  |
|  |  |  |  |  |  |  |  |  |  |  |  |  |  |  |

| **Table S8. Nodal efficiency among the RP-M (n=13), RP-R (n=12), and HC (n=50).** | | | | | | | | | | | | |  |
| --- | --- | --- | --- | --- | --- | --- | --- | --- | --- | --- | --- | --- | --- |
| **Index** | **ROIs** | **Coordinates** | | | **RP-M** | **RP-R** | **HC** | **1 vs 2 vs 3** | | **1 vs 3** | **2 vs 3** | **1 vs 2** |  |
|  |  |  |  |  |  |  |  | **p-unc** | **p-FDR** | **p-unc** | **p-unc** | **p-unc** |  |
| 16 | Left precuneus | -3 | -38.25 | 45 | 0.188(0.016) | 0.196(0.018) | 0.182(0.015) | 0.0085 | 0.2402 | 0.2515 | 0.0226 | 0.5728 |  |
| 33 | Left occipital | -2.0625 | -75 | 32.0625 | 0.190(0.016) | 0.189(0.022) | 0.179(0.017) | 0.0454 | 0.6053 | 0.0839 | 0.3486 | 1.0000 |  |
| 50 | Left posterior parietal | -35.0625 | -45.9375 | 48 | 0.159(0.027) | 0.176(0.021) | 0.178(0.015) | 0.0234 | 0.4304 | 0.0187 | 1.0000 | 1.0000 |  |
| 57 | Right ventral prefrontal cortex | 33.6 | 32.4 | 6.6 | 0.144(0.038) | 0.139(0.026) | 0.157(0.030) | 0.0415 | 0.6041 | 1.0000 | 0.0693 | 0.4740 |  |
| 59 | Right ventral frontal cortex | 51 | 23.0625 | 8.0625 | 0.163(0.025) | 0.163(0.025) | 0.177(0.018) | 0.0240 | 0.4304 | 0.1575 | 0.0585 | 1.0000 |  |
| 62 | Left anterior insula | -36 | 18 | 2.25 | 0.169(0.012) | 0.163(0.032) | 0.181(0.020) | 0.0043 | 0.1725 | 0.0842 | 0.0013 | 1.0000 |  |
| 69 | Right mid-Insula | 36.9375 | -2.0625 | -3 | 0.179(0.021) | 0.155(0.034) | 0.183(0.016) | 0.0014 | 0.1725 | 0.7701 | 0.0010 | 0.4755 |  |
| 70 | Left thalamus | -12 | -3 | 12.75 | 0.188(0.013) | 0.187(0.026) | 0.170(0.025) | 0.0090 | 0.2402 | 0.0069 | 0.4193 | 1.0000 |  |
| 96 | Left mid-insula | -42 | -3 | 11.25 | 0.168(0.028) | 0.148(0.052) | 0.177(0.019) | 0.0037 | 0.1725 | 0.4454 | 0.0030 | 1.0000 |  |
| 118 | Left temporal | -41.4 | -36.6 | 15.6 | 0.177(0.018) | 0.151(0.028) | 0.176(0.017) | 0.0022 | 0.1725 | 1.0000 | 0.0026 | 0.2215 |  |
| 138 | Left posterior occipital | -36.6 | -83.4 | -2.4 | 0.172(0.021) | 0.160(0.036) | 0.177(0.016) | 0.0328 | 0.5243 | 0.6063 | 0.0626 | 0.3140 |  |
| 145 | Left inferior cerebellum | -36.9375 | -54 | -36.9375 | 0.161(0.027) | 0.146(0.042) | 0.170(0.032) | 0.0242 | 0.4304 | 0.7612 | 0.0342 | 1.0000 |  |
| RP-M, RP-R, and HC are labeled as 1, 2, and 3, respectively; Comparison of degree centrality (AUC of Ne) among the 1, 2, and 3, ANCOVA (p<0.05) with covariates (age and sex). Significant results were found only at the uncorrected level.  **Note:** ANCOVA, Analysis of covariance; AUC, area under the curve; Data are mean (± standard deviation); False discovery rate; HC, Healthy controls; Ne, nodal efficiency; RP-M, Recovered patients with maintained; RP-R, Recovered patients with relapsed; | | | | | | | | | | | | |  |
|  |  |  |  |  |  |  |  |  |  |  |  |  |  |

| **Table S9. Hub nodes of the RP.** | | | | | | | |  | |
| --- | --- | --- | --- | --- | --- | --- | --- | --- | --- |
| **Index** | **Sub Network** | **ROIs** | **Coordinates** | | | **Degree** | |  | |
|  |  |  |  |  |  | **mean(±sd)** | |  | |
| 72 | CON | Right thalamus | 11.25 | -12 | 6 | 15.01 | (4.53) | |  |
| 16 | DMN | Left precuneus | -3 | -38.25 | 45 | 14.46 | (4.72) | |  |
| 105 | Sens | Right temporal | 59.4 | -12.6 | 8.4 | 14.40 | (4.26) | |  |
| 93 | Sens | Supplementary motor area | 0 | -0.9375 | 51.9375 | 14.33 | (4.08) | |  |
| 33 | DMN | Left occipital | -2.0625 | -75 | 32.0625 | 14.22 | (4.90) | |  |
| 133 | Occi | Right occipital | 15 | -77.0625 | 32.0625 | 14.20 | (4.90) | |  |
| 111 | Sens | Left temporal | -54 | -21.75 | 9 | 13.96 | (5.15) | |  |
| 135 | Occi | Left posterior occipital | -5.0625 | -80.0625 | 9 | 13.94 | (4.79) | |  |
| 41 | FPN | Left anterior cingulate cortex | -0.6 | 27.6 | 39.6 | 13.87 | (5.07) | |  |
| 126 | Occi | Right occipital | 18.9375 | -66 | -0.9375 | 13.84 | (3.92) | |  |
| 71 | CON | Left thalamus | -12 | -12 | 6 | 13.77 | (5.95) | |  |
| 157 | Cere | Right med cerebellum | 14.25 | -75 | -21 | 13.69 | (6.23) | |  |
| 64 | CON | Left intra-parietal sulcus | 0 | 15 | 45 | 13.61 | (4.28) | |  |
| 70 | CON | Left thalamus | -12 | -3 | 12.75 | 13.45 | (4.65) | |  |
| 131 | Occi | Left occipital | -15.9375 | -75.9375 | 33 | 13.28 | (4.33) | |  |
| 86 | CON | Left temporal | -59.4 | -47.4 | 11.4 | 13.22 | (5.17) | |  |
| 132 | Occi | Right occipital | 9 | -75.9375 | 14.0625 | 13.19 | (4.36) | |  |
| 156 | Cere | Right med cerebellum | 5.0625 | -75 | -11.0625 | 13.06 | (5.08) | |  |
| 58 | CON | Left anterior cingulate cortex | -2.25 | 30 | 27 | 12.91 | (4.70) | |  |
| 87 | CON | Left temporoparietal junction | -51.75 | -63 | 15 | 12.88 | (3.98) | |  |
| 30 | DMN | Left intra-parietal sulcus | -36 | -69 | 39.75 | 12.64 | (3.85) | |  |
| 28 | DMN | Light angular gyrus cortex | -48 | -63 | 35.25 | 12.47 | (3.76) | |  |
| 22 hubs were identified in the RP.  **Note**: CON, Cingulo-opercular network; Cere, Cerebellum network; DMN, Default mode network; FPN, Frontal parietal network; Occi, Occipital network; sd, standard deviation; Sens, Sensorimotor network; RP, Recovered patients. | | | | | | | |  | |
|  |  |  |  |  |  |  |  |  |  |
|  |  |  |  |  |  |  |  |  |  |
|  |  |  |  |  |  |  |  |  |  |

| **Table S10. Hub nodes of HC.** | | | | | | | |  |
| --- | --- | --- | --- | --- | --- | --- | --- | --- |
| **Index** | **Sub Network** | **ROIs** | **Coordinates** | | | **Degree** | |  |
|  |  |  |  |  |  | **mean(±sd)** | |  |
| 93 | Sens | Supplementary motor area | 0 | -0.9375 | 51.9375 | 14.10 | (3.94) |  |
| 113 | Sens | Right posterior insula | 42 | -24 | 17.25 | 13.90 | (5.50) |  |
| 105 | Sens | Right temporal | 59.4 | -12.6 | 8.4 | 13.58 | (4.38) |  |
| 87 | CON | Left temporoparietal junction | -51.75 | -63 | 15 | 13.30 | (4.29) |  |
| 72 | CON | Right thalamus | 11.25 | -12 | 6 | 13.17 | (5.37) |  |
| 135 | Occi | Left posterior occipital | -5.0625 | -80.0625 | 9 | 13.07 | (4.36) |  |
| 132 | Occi | Right occipital | 9 | -75.9375 | 14.0625 | 12.87 | (4.27) |  |
| 124 | Occi | Right temporal | 45.6 | -62.4 | 5.4 | 12.77 | (4.32) |  |
| 127 | Occi | Right occipital | 17.4 | -68.4 | 20.4 | 12.72 | (4.36) |  |
| 111 | Sens | Left temporal | -54 | -21.75 | 9 | 12.55 | (5.27) |  |
| 64 | CON | Left intra-parietal sulcus | 0 | 15 | 45 | 12.42 | (4.38) |  |
| 151 | Cere | Right lateral cerebellum | 21 | -63.9375 | -21.9375 | 12.36 | (4.48) |  |
| 8 | DMN | Right anterior cingulate cortex | 9 | 39 | 20.25 | 12.33 | (4.61) |  |
| 76 | CON | Left posterior insula | -30 | -27.75 | 9 | 12.33 | (4.77) |  |
| 133 | Occi | Right occipital | 15 | -77.0625 | 32.0625 | 12.29 | (3.55) |  |
| 154 | Cere | Left med cerebellum | -11.0625 | -72 | -14.0625 | 12.29 | (4.36) |  |
| 130 | Occi | Left occipital | -29.0625 | -75 | 27.9375 | 12.24 | (3.98) |  |
| 116 | Sens | Left parietal | -24 | -30 | 63.75 | 12.22 | (4.87) |  |
| 126 | Occi | Right occipital | 18.9375 | -66 | -0.9375 | 12.22 | (4.11) |  |
| 69 | CON | Right mid-insula | 36.9375 | -2.0625 | -3 | 12.17 | (4.01) |  |
| 157 | Cere | Right med cerebellum | 14.25 | -75 | -21 | 12.12 | (4.28) |  |
| 131 | Occi | Left occipital | -15.9375 | -75.9375 | 33 | 12.09 | (5.31) |  |
| 41 | FPN | Left anterior cingulate cortex | -0.6 | 27.6 | 39.6 | 12.07 | (5.09) |  |
| 23 hubs were identified in the HC.  **Note**: CON, Cingulo-opercular network; Cere, Cerebellum network; DMN, Default mode network; FPN, Frontal parietal network; HC, Healthy controls; Occi,Occipital network; sd, standard deviation; Sens, Sensorimotor network; | | | | | | | |  |
|  |  |  |  |  |  |  |  |  |
|  |  |  |  |  |  |  |  |  |
|  |  |  |  |  |  |  |  |  |

| **Table S11. Hub nodes of the RP-M.** | | | | | | | |  |
| --- | --- | --- | --- | --- | --- | --- | --- | --- |
| **Index** | **Sub Network** | **ROIs** | **Coordinates** | | | **Degree** | |  |
|  |  |  |  |  |  | **mean(±sd)** | |  |
| 72 | CON | Right thalamus | 11.25 | -12 | 6 | 14.893 | (4.155) |  |
| 41 | FPN | Left anterior cingulate cortex | -0.6 | 27.6 | 39.6 | 14.791 | (4.237) |  |
| 157 | Cere | Right med cerebellum | 14.25 | -75 | -21 | 14.579 | (5.980) |  |
| 133 | Occi | Right occipital | 15 | -77.0625 | 32.0625 | 14.575 | (3.817) |  |
| 135 | Occi | Left posterior occipital | -5.0625 | -80.0625 | 9 | 14.466 | (3.862) |  |
| 71 | CON | Left thalamus | -12 | -12 | 6 | 14.150 | (4.644) |  |
| 64 | CON | Left intra-parietal sulcus | 0 | 15 | 45 | 14.114 | (3.870) |  |
| 33 | DMN | Left occipital | -2.0625 | -75 | 32.0625 | 14.029 | (4.093) |  |
| 93 | Sens | Supplementary motor area | 0 | -0.9375 | 51.9375 | 14.005 | (4.107) |  |
| 111 | Sens | Left temporal | -54 | -21.75 | 9 | 13.823 | (4.106) |  |
| 16 | DMN | Left precuneus | -3 | -38.25 | 45 | 13.781 | (4.385) |  |
| 58 | CON | Left anterior cingulate cortex | -2.25 | 30 | 27 | 13.778 | (4.629) |  |
| 159 | Cere | Left inferior cerebellum | -6 | -78.75 | -33 | 13.712 | (4.656) |  |
| 126 | Occi | Right occipital | 18.9375 | -66 | -0.9375 | 13.691 | (3.604) |  |
| 70 | CON | Left thalamus | -12 | -3 | 12.75 | 13.516 | (3.413) |  |
| 156 | Cere | Right med cerebellum | 5.0625 | -75 | -11.0625 | 13.514 | (4.329) |  |
| 131 | Occi | Left occipital | -15.9375 | -75.9375 | 33 | 13.508 | (3.791) |  |
| 30 | DMN | Left intra-parietal sulcus | -36 | -69 | 39.75 | 13.505 | (3.812) |  |
| 105 | Sens | Right temporal | 59.4 | -12.6 | 8.4 | 13.428 | (5.042) |  |
| 63 | CON | Left basal ganglia | -6 | 17.0625 | 33.9375 | 13.212 | (3.921) |  |
| 116 | Sens | Left parietal | -24 | -30 | 63.75 | 13.211 | (4.259) |  |
| 132 | Occi | Right occipital | 9 | -75.9375 | 14.0625 | 13.139 | (3.949) |  |
| 3 | DMN | Left anterior prefrontal cortex | -24.75 | 51 | 27 | 12.813 | (5.047) |  |
| 87 | CON | Left temporoparietal junction | -51.75 | -63 | 15 | 12.753 | (3.010) |  |
| 142 | Occi | Left posterior occipital | -3.9375 | -93.9375 | 12 | 12.728 | (2.932) |  |
| 154 | Cere | Left med cerebellum | -11.0625 | -72 | -14.0625 | 12.683 | (3.667) |  |
| 22 | DMN | Right precuneus | 5.0625 | -50.0625 | 33 | 12.678 | (3.055) |  |
| 27 hubs were identified in the RP-M.  **Note:** CON, Cingulo-opercular network; Cere, Cerebellum network; DMN, Default mode network; FPN, Frontal parietal network; Occi, Occipital network; sd, standard deviation; Sens, Sensorimotor network; RP-M, Recovered patients with maintained. | | | | | | | |  |
|  |  |  |  |  |  |  |  |  |
|  |  |  |  |  |  |  |  |  |
|  |  |  |  |  |  |  |  |  |

| **Table S12. Hub nodes of the RP-R.** | | | | | | | |  |
| --- | --- | --- | --- | --- | --- | --- | --- | --- |
| **Index** | **Sub Net** | **ROIs** | **Coordinates** | | | **Degree** | |  |
|  |  |  |  |  |  | **mean(±sd)** | |  |
| 16 | DMN | Left precuneus | -3 | -38.25 | 45 | 15.990 | (5.322) |  |
| 111 | Sens | Left temporal | -54 | -21.75 | 9 | 15.485 | (6.162) |  |
| 105 | Sens | Right temporal | 59.4 | -12.6 | 8.4 | 15.035 | (3.904) |  |
| 72 | CON | Right thalamus | 11.25 | -12 | 6 | 14.931 | (4.469) |  |
| 33 | DMN | Left occipital | -2.0625 | -75 | 32.0625 | 14.498 | (5.871) |  |
| 86 | CON | Left temporal | -59.4 | -47.4 | 11.4 | 14.380 | (5.211) |  |
| 93 | Sens | Supplementary motor area | 0 | -0.9375 | 51.9375 | 14.234 | (4.307) |  |
| 135 | Occi | Left posterior occipital | -5.0625 | -80.0625 | 9 | 14.215 | (6.093) |  |
| 41 | FPN | Left anterior cingulate cortex | -0.6 | 27.6 | 39.6 | 14.157 | (6.211) |  |
| 70 | CON | Left thalamus | -12 | -3 | 12.75 | 14.078 | (5.977) |  |
| 133 | Occi | Right occipital | 15 | -77.0625 | 32.0625 | 13.806 | (5.775) |  |
| 126 | Occi | Right occipital | 18.9375 | -66 | -0.9375 | 13.797 | (4.183) |  |
| 150 | Cere | Left med cerebellum | -15.9375 | -63.9375 | -21 | 13.549 | (5.451) |  |
| 132 | Occi | Right occipital | 9 | -75.9375 | 14.0625 | 13.443 | (5.369) |  |
| 75 | CON | Right basal ganglia | 11.0625 | -24 | 2.0625 | 13.391 | (4.860) |  |
| 58 | CON | Left anterior cingulate cortex | -2.25 | 30 | 27 | 13.288 | (5.098) |  |
| 71 | CON | Left thalamus | -12 | -12 | 6 | 13.260 | (5.984) |  |
| 129 | Occi | Right occipital | 29.4 | -72.6 | 29.4 | 13.253 | (5.948) |  |
| 127 | Occi | Right occipital | 17.4 | -68.4 | 20.4 | 13.167 | (4.487) |  |
| 8 | DMN | Right anterior cingulate cortex | 9 | 39 | 20.25 | 13.159 | (5.146) |  |
| 64 | CON | Left intra-parietal sulcus | 0 | 15 | 45 | 13.064 | (5.136) |  |
| 67 | CON | Right basal ganglia | 14.0625 | 6 | 6.9375 | 13.023 | (4.737) |  |
| 87 | CON | Left temporoparietal junction | -51.75 | -63 | 15 | 12.976 | (4.979) |  |
| 28 | DMN | Right angular gyrus cortex | -48 | -63 | 35.25 | 12.875 | (4.565) |  |
| 27 | DMN | Right angular gyrus cortex | 51 | -59.0625 | 33.9375 | 12.860 | (4.178) |  |
| 131 | Occi | Left occipital | -15.9375 | -75.9375 | 33 | 12.823 | (5.534) |  |
| 26 hubs were identified in the RP-R.  **Note:** CON, Cingulo-opercular network; Cere, Cerebellum network; DMN, Default mode network; FPN, Frontal parietal network; Occi, Occipital network; sd, standard deviation; Sens, Sensorimotor network; RP-R, Recovered patients with relapsed. | | | | | | | |  |
|  |  |  |  |  |  |  |  |  |
|  |  |  |  |  |  |  |  |  |
|  |  |  |  |  |  |  |  |  |

| **Table S13a. FC in significantly different connected components between the RP (n=30) and HC (n=50).** | | | | | | | |
| --- | --- | --- | --- | --- | --- | --- | --- |
| **Node index** | | **Node label** | **RP** | | **HC** | | **t value** |
| **"i "** | **" j"** | **"i to j"** | **mean(±sd)** | | **mean(±sd)** | |
| **Connected component 1 (p=0.031)** | | | **0.388** | **(0.042)** | **0.166** | **(0.046)** |  |
| 65 | 66 | Left ventral frontal cortex to Left basal ganglia. | 0.395 | (0.200) | 0.206 | (0.189) | 4.25 |
| 66 | 86 | Left basal ganglia to Left temporal. | 0.427 | (0.196) | 0.220 | (0.236) | 3.98 |
| 70 | 86 | Left thalamus to Left temporal. | 0.414 | (0.228) | 0.153 | (0.250) | 4.67 |
| 70 | 87 | Left thalamus to Left temporoparietal junction. | 0.399 | (0.244) | 0.164 | (0.192) | 4.69 |
| 70 | 131 | Left thalamus to Left occipital. | 0.308 | (0.229) | 0.089 | (0.215) | 4.20 |
| NBS analysis between the connectomes of the RP and HC, two-sample t-test (p<0.05 and 3.6≤t≤4.2) with 10000 permutations and covariates (age and sex). At the median threshold t=3.9, RP had significantly higher FC of a connected component compared to HC.  **Note**: FC, Functional connectivity; HC, Healthy controls; RP, Recovered patients; sd, standard deviation; | | | | | | | |
|

| **Table S13b. Nodal strength in significantly different connected components between the RP (n=30) and HC (n=50).** | | | | | | | | | | |  |
| --- | --- | --- | --- | --- | --- | --- | --- | --- | --- | --- | --- |
| **Node index** | **Node label** | **Coordinate** | | | **RP** | | **HC** | | **p-unc** | **p-bonf** |  |
|  |  |  |  |  | **mean(±sd)** | | **mean(±sd)** | |  |  |  |
| 65 | Left ventral frontal cortex | -45.6 | 9.6 | 14.4 | 0.394 | (0.200) | 0.234 | (0.145) | <0.0001 | 0.0003 |  |
| 66 | Left basal ganglia | -20.0625 | 6 | 6.9375 | 0.820 | (0.346) | 0.506 | (0.254) | <0.0001 | <0.0001 |  |
| 70 | Left thalamus | -12 | -3 | 12.75 | 1.147 | (0.555) | 0.600 | (0.369) | <0.0001 | <0.0001 |  |
| 86 | Left temporal | -59.4 | -47.4 | 11.4 | 0.840 | (0.393) | 0.499 | (0.341) | <0.0001 | 0.0001 |  |
| 87 | Left temporoparietal junction | -51.75 | -63 | 15 | 0.406 | (0.232) | 0.199 | (0.157) | <0.0001 | <0.0001 |  |
| 131 | Left occipital | -15.9375 | -75.9375 | 33 | 0.326 | (0.201) | 0.174 | (0.138) | <0.0001 | 0.0001 |  |
| Nodal strength comparison between the RP and HC at the median threshold t=3.9  **Note:** Nodal strength was calculated by absolute values of FC; FC, Functional connectivity; HC, Healthy controls; p-unc, uncorrected p-value; p-bonf, Bonferroni corrected p-value (n= node number of a significant component); RP, Recovered patients; sd, standard deviation; | | | | | | | | | | |  |
|  |  |  |  |  |  |  |  |  |  |  |  |
|  |  |  |  |  |  |  |  |  |  |  |  |

| **Table S14a. FC in significantly different connected components between the RP (n=30) and HC (n=50).** | | | | | | | |
| --- | --- | --- | --- | --- | --- | --- | --- |
| **Node index** | | **Node label** | **RP** | | **HC** | | **t value** |
| **"i "** | **" j"** | **"i to j"** | **mean(±sd)** | | **mean(±sd)** | |
| **Connected component 1 (p=0.022)** | | | **0.373** | **(0.047)** | **0.135** | **(0.033)** |  |
| 70 | 86 | Left thalamus to Left temporal. | 0.414 | (0.228) | 0.153 | (0.250) | 4.67 |
| 70 | 87 | Left thalamus to Left temporoparietal junction. | 0.399 | (0.244) | 0.164 | (0.192) | 4.69 |
| 70 | 131 | Left thalamus to Left occipital. | 0.308 | (0.229) | 0.089 | (0.215) | 4.20 |
| NBS analysis between connectomes of the RP and HC, two-sample t-test (p<0.05 and 3.6≤t≤4.2) with 10000 permutations and covariates (age and sex). At the maximum threshold t=4.2, RP had significantly higher FC of a connected component compared to HC.  Note: FC, Functional connectivity; HC, Healthy controls; RP, Recovered patients; sd, standard deviation; | | | | | | | |
|

| **Table S14b. Nodal strength in significantly different connected components between the RP (n=30) and HC (n=50).** | | | | | | | | | | |
| --- | --- | --- | --- | --- | --- | --- | --- | --- | --- | --- |
| **Node index** | **Node label** | **Coordinate** | | | **RP** | | **HC** | | **p-unc** | **p-bonf** |
|  |  |  |  |  | **mean(±sd)** | | **mean(±sd)** | |  |  |
| 70 | Left thalamus | -12 | -3 | 12.75 | 1.147 | (0.555) | 0.600 | (0.369) | <0.0001 | <0.0001 |
| 86 | Left temporal | -59.4 | -47.4 | 11.4 | 0.415 | (0.228) | 0.227 | (0.184) | 0.0001 | 0.0005 |
| 87 | Left temporoparietal junction | -51.75 | -63 | 15 | 0.406 | (0.232) | 0.199 | (0.157) | 0.0000 | 0.0000 |
| 131 | Left occipital | -15.9375 | -75.9375 | 33 | 0.326 | (0.201) | 0.174 | (0.138) | 0.0001 | 0.0005 |
| Nodal strength comparison between RP and HC at the maximum threshold t=4.2  **Note:** Nodal strength was calculated by absolute values of FC; FC, Functional connectivity; HC, Healthy controls; p-unc, uncorrected p-value; p-bonf, Bonferroni corrected p-value (n= node number of a significant component); RP, Recovered patients; sd, standard deviation; | | | | | | | | | | |
|  |  |  |  |  |  |  |  |  |  |  |

| **Table S15. FC in significantly different connected components among the RP-M (n=13), RP-R (n=12), and HC (n=50).** | | | | | | | | | |  |
| --- | --- | --- | --- | --- | --- | --- | --- | --- | --- | --- |
| **Node index** | | **Node label** | **RP-M** | | **RP-R** | | **HC** | | **F value** |  |
| **"i "** | **" j"** | **"i to j"** | **mean(±sd)** | | **mean(±sd)** | | **mean(±sd)** | |  |
| **Connected component 1 (p=0.043)** | | | **0.202** | **(0.149)** | **0.413** | **(0.101)** | **0.167** | **(0.084)** |  |  |
| 8 | 11 | Right anterior cingulate cortex to Left superior frontal cortex. | 0.395 | (0.235) | 0.653 | (0.332) | 0.346 | (0.251) | 8.41 |  |
| 14 | 32 | Right posterior cingulate cortex to Right occipital. | 0.265 | (0.213) | 0.550 | (0.330) | 0.170 | (0.250) | 9.64 |  |
| 15 | 37 | Right fusiform to Right ventral anterior prefrontal cortex. | -0.055 | (0.176) | 0.264 | (0.167) | 0.161 | (0.211) | 8.25 |  |
| 1 | 38 | Right ventral medial prefrontal cortex to Left ventral anterior prefrontal cortex. | 0.197 | (0.330) | 0.365 | (0.311) | 0.054 | (0.266) | 7.66 |  |
| 4 | 38 | Right ventral medial prefrontal cortex to left ventral anterior prefrontal cortex. | -0.030 | (0.306) | 0.378 | (0.293) | 0.024 | (0.304) | 8.71 |  |
| 25 | 38 | Left precuneus to Left ventral anterior prefrontal cortex. | 0.148 | (0.262) | 0.332 | (0.254) | 0.010 | (0.276) | 8.52 |  |
| 33 | 38 | Left occipital to Left ventral anterior prefrontal cortex. | 0.190 | (0.233) | 0.424 | (0.195) | 0.078 | (0.232) | 12.26 |  |
| 33 | 41 | Left occipital to Left anterior cingulate cortex. | 0.355 | (0.265) | 0.453 | (0.202) | 0.129 | (0.240) | 10.63 |  |
| 8 | 43 | Right anterior cingulate cortex to Left ventral prefrontal cortex. | 0.085 | (0.171) | 0.433 | (0.271) | 0.200 | (0.197) | 8.34 |  |
| 14 | 43 | Right posterior cingulate cortex to Left ventral prefrontal cortex. | 0.138 | (0.240) | 0.342 | (0.216) | 0.067 | (0.193) | 8.60 |  |
| 1 | 51 | Right ventral medial prefrontal cortex to Left inferior parietal lobe. | 0.116 | (0.266) | 0.338 | (0.260) | 0.032 | (0.247) | 8.01 |  |
| 5 | 55 | Left ventral medial prefrontal cortex to Right intra-parietal sulcus. | -0.004 | (0.275) | 0.279 | (0.227) | -0.015 | (0.234) | 7.79 |  |
| 13 | 55 | Left inferior temporal to Right intra-parietal sulcus. | 0.254 | (0.177) | 0.363 | (0.271) | 0.074 | (0.242) | 8.26 |  |
| 33 | 55 | Left occipital to Right intra-parietal sulcus. | 0.202 | (0.329) | 0.555 | (0.241) | 0.211 | (0.247) | 7.89 |  |
| 65 | 66 | Left ventral frontal cortex to Left basal ganglia. | 0.385 | (0.185) | 0.466 | (0.205) | 0.206 | (0.189) | 11.82 |  |
| 65 | 67 | Left ventral frontal cortex to Right basal ganglia. | 0.401 | (0.176) | 0.478 | (0.245) | 0.234 | (0.212) | 8.86 |  |
| 16 | 70 | Left precuneus to Left thalamus. | 0.386 | (0.202) | 0.523 | (0.214) | 0.290 | (0.192) | 8.69 |  |
| 65 | 70 | Left ventral frontal cortex to Left thalamus. | 0.399 | (0.214) | 0.479 | (0.191) | 0.259 | (0.190) | 7.51 |  |
| 10 | 76 | Right superior frontal cortex to Left posterior insula. | 0.014 | (0.159) | 0.351 | (0.243) | 0.172 | (0.209) | 9.49 |  |
| 15 | 76 | Right fusiform to Left posterior insula. | 0.162 | (0.207) | 0.412 | (0.203) | 0.272 | (0.188) | 8.37 |  |
| 70 | 77 | Left thalamus to Right temporal. | 0.364 | (0.262) | 0.425 | (0.186) | 0.159 | (0.244) | 7.74 |  |
| 41 | 86 | Left anterior cingulate cortex to Left temporal. | 0.231 | (0.298) | 0.485 | (0.236) | 0.181 | (0.245) | 7.46 |  |
| 66 | 86 | Left basal ganglia to Left temporal. | 0.415 | (0.204) | 0.476 | (0.205) | 0.220 | (0.236) | 7.94 |  |
| 67 | 86 | Right basal ganglia to Left temporal. | 0.342 | (0.211) | 0.523 | (0.276) | 0.204 | (0.221) | 9.47 |  |
| 70 | 86 | Left thalamus to Left temporal. | 0.372 | (0.221) | 0.501 | (0.200) | 0.153 | (0.250) | 12.01 |  |
| 72 | 86 | Right thalamus to Left temporal. | 0.372 | (0.202) | 0.528 | (0.236) | 0.241 | (0.219) | 8.27 |  |
| 14 | 87 | Right posterior cingulate cortex to Left temporoparietal junction. | 0.159 | (0.255) | 0.557 | (0.337) | 0.187 | (0.221) | 9.64 |  |
| 70 | 87 | Left thalamus to Left temporoparietal junction. | 0.361 | (0.273) | 0.473 | (0.245) | 0.164 | (0.192) | 11.29 |  |
| 67 | 90 | Right basal ganglia to Left ventral prefrontal cortex. | 0.242 | (0.103) | 0.444 | (0.244) | 0.191 | (0.204) | 8.02 |  |
| 37 | 109 | Right ventral anterior prefrontal cortex to Left parietal. | -0.033 | (0.192) | 0.158 | (0.236) | 0.251 | (0.218) | 8.28 |  |
| 67 | 119 | Right basal ganglia to Left temporal. | 0.368 | (0.204) | 0.433 | (0.264) | 0.194 | (0.190) | 9.13 |  |
| 70 | 119 | Left thalamus to Left temporal. | 0.368 | (0.199) | 0.409 | (0.264) | 0.166 | (0.229) | 7.52 |  |
| 14 | 121 | Right basal ganglia to Left temporal. | 0.163 | (0.174) | 0.285 | (0.272) | -0.002 | (0.249) | 7.51 |  |
| 41 | 133 | Left anterior cingulate cortex to Right occipital. | 0.329 | (0.168) | 0.363 | (0.316) | 0.101 | (0.229) | 7.99 |  |
| 46 | 143 | Right dorsal prefrontal cortex to Left lateral cerebellum | -0.015 | (0.243) | 0.283 | (0.178) | 0.190 | (0.220) | 7.99 |  |
| 50 | 143 | Left posterior parietal to Left lateral cerebellum. | 0.118 | (0.241) | 0.427 | (0.260) | 0.226 | (0.192) | 8.54 |  |
| 54 | 143 | Left intra-parietal sulcus to Left lateral cerebellum. | -0.018 | (0.154) | 0.295 | (0.145) | 0.193 | (0.175) | 12.11 |  |
| 55 | 143 | Right intra-parietal sulcus to Left lateral cerebellum. | 0.018 | (0.190) | 0.335 | (0.261) | 0.214 | (0.180) | 9.09 |  |
| 15 | 144 | Right fusiform to Left lateral cerebellum. | 0.284 | (0.222) | 0.556 | (0.209) | 0.308 | (0.221) | 7.92 |  |
| 46 | 144 | Right dorsal prefrontal cortex to Left lateral cerebellum. | 0.019 | (0.167) | 0.251 | (0.227) | 0.196 | (0.176) | 7.87 |  |
| 65 | 149 | Left ventral frontal cortex to Right inferior cerebellum. | 0.248 | (0.170) | 0.477 | (0.215) | 0.218 | (0.181) | 9.33 |  |
| 15 | 150 | Right fusiform to Left medial cerebellum. | 0.132 | (0.252) | 0.493 | (0.216) | 0.247 | (0.201) | 11.75 |  |
| 44 | 150 | Left dorsolateral prefrontal cortex to Left medial cerebellum. | 0.045 | (0.186) | 0.391 | (0.192) | 0.181 | (0.203) | 9.60 |  |
| NBS analysis among the connectomes of the RP-M, RP-R and HC, ANCOVA (age and sex) with 10000 permutations (p<0.05 and 7.0≤F≤7.8). At the median threshold F=7.4, a significantly different connected component was detected (43 edges between 41 nodes) among three groups.  **Note:** ANCOVA, Covariance analysis; FC, Functional connectivity; HC, Healthy controls; RP-M, Recovered patients with maintained; RP-R, Recovered patients with relapsed; sd, standard deviation; | | | | | | | | | |  |
|  |
|  |
|  |

| **Table S16. Comparison of altered connected components between the RP-M (n=13) and HC (n=50).** | | | | | |  |
| --- | --- | --- | --- | --- | --- | --- |
| **Node index** | | **Node label** | **RP-M** | **HC** | **t value** |  |
| **"i "** | **" j"** | **"i to j"** | **mean(±sd)** | **mean(±sd)** |  |
| No significant | | | | | |  |
| Posthoc test between the connectomes of the RP-M and HC, (p<0.05/3 and 3.6≤t≤4.2) with 10000 permutations and covariates (age and sex).  **Note:** HC, Healthy controls; RP-M, Recovered patients with maintained; sd, standard deviation; | | | | | |  |
|  |

| **Table S17a. Comparison of altered connected components between the RP-R (n=12) and HC (n=50).** | | | | | | | |  |
| --- | --- | --- | --- | --- | --- | --- | --- | --- |
| **Node index** | | **Node label** | **RP-R** | | **HC** | | **t value** |  |
| **"i "** | **" j"** | **"i to j"** | **mean(±sd)** | | **mean(±sd)** | |  |
| **Connected component 1 (p=0.036)** | | | **0.653** | **(0.000)** | **0.346** | **(0.000)** |  |  |
| 8 | 11 | Right anterior cingulate cortex to Left superior frontal cortex. | 0.653 | (0.332) | 0.346 | (0.251) | 3.99 |  |
| **Connected component 2 (p<0.001)** | | | **0.437** | **(0.085)** | **0.132** | **(0.083)** |  |  |
| 14 | 32 | Right posterior cingulate cortex to Right occipital. | 0.550 | (0.330) | 0.170 | (0.250) | 4.07 |  |
| 1 | 38 | Right ventral medial prefrontal cortex to Left ventral anterior prefrontal cortex. | 0.365 | (0.311) | 0.054 | (0.266) | 3.99 |  |
| 4 | 38 | Right ventral medial prefrontal cortex to left ventral anterior prefrontal cortex. | 0.378 | (0.293) | 0.024 | (0.304) | 3.85 |  |
| 25 | 38 | Left precuneus to Left ventral anterior prefrontal cortex. | 0.332 | (0.254) | 0.010 | (0.276) | 3.84 |  |
| 33 | 38 | Left occipital to Left ventral anterior prefrontal cortex. | 0.424 | (0.195) | 0.078 | (0.232) | 4.77 |  |
| 33 | 41 | Left occipital to Left anterior cingulate cortex. | 0.453 | (0.202) | 0.129 | (0.240) | 4.12 |  |
| 14 | 43 | Right posterior cingulate cortex to Left ventral prefrontal cortex. | 0.342 | (0.216) | 0.067 | (0.193) | 4.17 |  |
| 1 | 51 | Right ventral medial prefrontal cortex to Left inferior parietal lobe. | 0.338 | (0.260) | 0.032 | (0.247) | 4.09 |  |
| 5 | 55 | Left ventral medial prefrontal cortex to Right intra-parietal sulcus. | 0.279 | (0.227) | -0.015 | (0.234) | 4.08 |  |
| 13 | 55 | Left inferior temporal to Right intra-parietal sulcus. | 0.363 | (0.271) | 0.074 | (0.242) | 3.63 |  |
| 33 | 55 | Left occipital to Right intra-parietal sulcus. | 0.555 | (0.241) | 0.211 | (0.247) | 4.23 |  |
| 16 | 70 | Left precuneus to Left thalamus. | 0.523 | (0.214) | 0.290 | (0.192) | 3.93 |  |
| 41 | 86 | Left anterior cingulate cortex to Left temporal. | 0.485 | (0.236) | 0.181 | (0.245) | 3.83 |  |
| 67 | 86 | Right basal ganglia to Left temporal. | 0.523 | (0.276) | 0.204 | (0.221) | 3.97 |  |
| 70 | 86 | Left thalamus to Left temporal. | 0.501 | (0.200) | 0.153 | (0.250) | 4.15 |  |
| 72 | 86 | Right thalamus to Left temporal. | 0.528 | (0.236) | 0.241 | (0.219) | 3.74 |  |
| 14 | 87 | Right posterior cingulate cortex to Left temporoparietal junction. | 0.557 | (0.337) | 0.187 | (0.221) | 3.74 |  |
| 70 | 87 | Left thalamus to Left temporoparietal junction. | 0.361 | (0.273) | 0.164 | (0.192) | 4.27 |  |
| 67 | 90 | Right basal ganglia to Left ventral prefrontal cortex. | 0.444 | (0.244) | 0.191 | (0.204) | 3.74 |  |
| 67 | 119 | Right basal ganglia to Left temporal. | 0.433 | (0.264) | 0.194 | (0.190) | 3.88 |  |
| **Connected component 3 (p=0.0012)** | | | **0.525** | **(0.031)** | **0.278** | **(0.030)** |  |  |
| 15 | 144 | Right fusiform to Left lateral cerebellum. | 0.556 | (0.209) | 0.308 | (0.221) | 3.68 |  |
| 15 | 150 | Right fusiform to Left medial cerebellum. | 0.493 | (0.216) | 0.247 | (0.201) | 3.97 |  |
| **Connected component 4 (p=0.0012)** | | | **0.471** | **(0.005)** | **0.212** | **(0.006)** |  |  |
| 65 | 66 | Left ventral frontal cortex to Left basal ganglia. | 0.466 | (0.205) | 0.206 | (0.189) | 4.07 |  |
| 65 | 149 | Left ventral frontal cortex to Right inferior cerebellum. | 0.477 | (0.215) | 0.218 | (0.181) | 4.18 |  |
| Posthoc test between connectomes of RP-R and HC, (p<0.05/3 and 3.0≤t≤4.2) with 10000 permutations and covariates (age and sex)  at the median threshold t=3.6, RP-R had significantly higher FC of four connected components than HC.  **Note:** FC, Functional connectivity; HC, Healthy controls; RP-R, Recovered patients with relapsed; sd, standard deviation; | | | | | | | |  |
|  |
|  |
|  |
|  |

| **Table S17b. Nodal strength in significantly different connected components between the RP-R (n=12) and HC (n=50).** | | | | | | | | | | |
| --- | --- | --- | --- | --- | --- | --- | --- | --- | --- | --- |
| **Node index** | **Node label** | **Coordinate** | | | **RP-R** | | **HC** | | **p-unc** | **p-bonf** |
|  |  |  |  |  | **mean(±sd)** | | **mean(±sd)** | |  |  |
| 1 | Right ventral medial prefrontal cortex | 6 | 63.75 | 3 | 0.710 | (0.517) | 0.409 | (0.270) | 0.0062 | 0.1312 |
| 4 | Right ventral medial prefrontal cortex | 9 | 51 | 15.75 | 0.393 | (0.268) | 0.241 | (0.173) | 0.0183 | 0.3838 |
| 5 | Left ventral medial prefrontal cortex | -6 | 50.0625 | -0.9375 | 0.294 | (0.204) | 0.165 | (0.161) | 0.0217 | 0.4562 |
| 8 | Right anterior cingulate cortex | 9 | 39 | 20.25 | 0.653 | (0.332) | 0.367 | (0.218) | 0.0005 | 0.0011 |
| 11 | Left superior frontal cortex | -15.9375 | 29.0625 | 54 | 0.653 | (0.332) | 0.367 | (0.218) | 0.0005 | 0.0011 |
| 13 | Left inferior temporal | -59.0625 | -24.9375 | -15 | 0.364 | (0.271) | 0.198 | (0.155) | 0.0061 | 0.1280 |
| 14 | Right posterior cingulate cortex | 0.6 | -26.4 | 30.6 | 1.475 | (0.706) | 0.644 | (0.321) | <0.0001 | <0.0001 |
| 15 | Left ventral medial prefrontal cortex | 27.9375 | -36.9375 | -15 | 1.049 | (0.381) | 0.601 | (0.334) | 0.0001 | 0.0004 |
| 16 | Left precuneus | -3 | -38.25 | 45 | 0.524 | (0.214) | 0.304 | (0.174) | 0.0004 | 0.0086 |
| 25 | Left precuneus | -6 | -56.0625 | 29.0625 | 0.332 | (0.254) | 0.218 | (0.155) | 0.0494 | 1.0000 |
| 32 | Right occipital | 45 | -72 | 29.25 | 0.562 | (0.312) | 0.246 | (0.177) | <0.0001 | 0.0003 |
| 33 | Left occipital | -2.0625 | -75 | 32.0625 | 1.434 | (0.428) | 0.665 | (0.310) | <0.0001 | <0.0001 |
| 38 | Left ventral anterior prefrontal cortex | -42.6 | 47.4 | 2.4 | 1.520 | (0.783) | 0.879 | (0.442) | 0.0003 | 0.0065 |
| 41 | Left anterior cingulate cortex | -0.6 | 27.6 | 39.6 | 0.937 | (0.396) | 0.445 | (0.270) | <0.0001 | 0.0001 |
| 43 | Left ventral prefrontal cortex | -51.6 | 27.6 | 17.4 | 0.357 | (0.189) | 0.162 | (0.129) | 0.0001 | 0.0014 |
| 51 | Left inferior parietal lobe | -48 | -47.0625 | 48.9375 | 0.341 | (0.256) | 0.191 | (0.160) | 0.0128 | 0.2680 |
| 55 | Right intra-parietal sulcus | 32.4 | -59.4 | 41.4 | 1.212 | (0.598) | 0.618 | (0.336) | <0.0001 | 0.0004 |
| 65 | Left ventral frontal cortex | -45.6 | 9.6 | 14.4 | 0.941 | (0.394) | 0.459 | (0.239) | <0.0001 | <0.0001 |
| 66 | Left basal ganglia | -20.0625 | 6 | 6.9375 | 0.464 | (0.205) | 0.234 | (0.145) | <0.0001 | 0.0001 |
| 67 | Right basal ganglia | 14.0625 | 6 | 6.9375 | 1.404 | (0.677) | 0.690 | (0.355) | <0.0001 | 0.0001 |
| 70 | Left thalamus | -12 | -3 | 12.75 | 1.499 | (0.549) | 0.731 | (0.347) | <0.0001 | <0.0001 |
| 72 | Right thalamus | 11.25 | -12 | 6 | 0.529 | (0.237) | 0.270 | (0.177) | 0.0001 | 0.0016 |
| 86 | Left temporal | -59.4 | -47.4 | 11.4 | 2.037 | (0.852) | 0.971 | (0.618) | <0.0001 | 0.0001 |
| 87 | Left temporoparietal junction | -51.75 | -63 | 15 | 1.030 | (0.454) | 0.435 | (0.240) | <0.0001 | <0.0001 |
| 90 | Left ventral prefrontal cortex | -54.6 | 6.6 | 23.4 | 0.447 | (0.235) | 0.228 | (0.158) | 0.0003 | 0.0053 |
| 119 | Left temporal | -53.4 | -36.6 | 12.6 | 0.434 | (0.262) | 0.225 | (0.144) | 0.0004 | 0.0074 |
| 144 | Left lateral cerebellum | -24 | -54 | -21 | 0.556 | (0.207) | 0.330 | (0.194) | 0.0007 | 0.0020 |
| 149 | Right inferior cerebellum | 32.4 | -60.6 | -30.6 | 0.477 | (0.215) | 0.225 | (0.161) | <0.0001 | 0.0001 |
| 150 | Left medial cerebellum | -15.9375 | -63.9375 | -21 | 0.493 | (0.217) | 0.272 | (0.166) | 0.0002 | 0.0007 |
| Nodal strength comparison between RP-R and HC at the median threshold t=3.6  **Note:** Nodal strength was calculated by absolute values of FC; FC, Functional connectivity; HC, Healthy controls; p-unc, uncorrected p-value; p-bonf, Bonferroni corrected p-value (n= node number of a significant component); RP-R, Recovered patients with relapsed; sd, standard deviation; | | | | | | | | | | |
|  |  |  |  |  |  |  |  |  |  |  |
|  |  |  |  |  |  |  |  |  |  |  |
|  |  |  |  |  |  |  |  |  |  |  |

| **Table S18a. Comparison of altered connected component between the RP-R (n=12) and HC (n=50).** | | | | | | | |  |
| --- | --- | --- | --- | --- | --- | --- | --- | --- |
| **Node index** | | **Node label** | **RP-R** | | **HC** | | **t value** |  |
| **"i "** | **" j"** | **"i to j"** | **mean(±sd)** | | **mean(±sd)** | |  |
| **Connected component 1 (p=0.009)** | | | **0.490** | **(0.065)** | **0.145** | **(0.067)** |  |  |
| 33 | 38 | Left occipital to Left ventral anterior prefrontal cortex. | 0.424 | (0.195) | 0.078 | (0.232) | 4.77 |  |
| 33 | 55 | Left occipital to Right intra-parietal sulcus. | 0.555 | (0.241) | 0.211 | (0.247) | 4.23 |  |
| **Connected component 2 (p=0.009)** | | | **0.260** | **(0.101)** | **0.176** | **(0.012)** |  |  |
| 14 | 87 | Right posterior cingulate cortex to Left temporoparietal junction. | 0.159 | (0.255) | 0.187 | (0.221) | 3.74 |  |
| 70 | 87 | Left thalamus to Left temporoparietal junction. | 0.361 | (0.273) | 0.164 | (0.192) | 4.27 |  |
| Posthoc test between connectomes of the RP-R and HC, (p<0.05/3 and 3.0≤t≤4.2) with 10000 permutations and covariates (age and sex)  at the maximum threshold t=4.2, RP-R had significantly higher FC of two connected components than HC.  **Note:** FC, Functional connectivity; HC, Healthy controls; RP-R, Recovered patients with relapsed; sd, standard deviation; | | | | | | | |  |
|  |
|  |
|  |

| **Table S18b. Nodal strength in significantly different connected components between the RP-R (n=12) and HC (n=50).** | | | | | | | | | | |  |
| --- | --- | --- | --- | --- | --- | --- | --- | --- | --- | --- | --- |
| **Node index** | **Node label** | **Coordinate** | | | **RP-R** | | **HC** | | **p-unc** | **p-bonf** |  |
|  |  |  |  |  | **mean(±sd)** | | **mean(±sd)** | |  |  |  |
| 14 | Right posterior cingulate cortex | 0.6 | -26.4 | 30.6 | 0.236 | (0.164) | 0.236 | (0.164) | <0.0001 | <0.0001 |  |
| 33 | Left occipital | -2.0625 | -75 | 32.0625 | 0.456 | (0.237) | 0.456 | (0.237) | <0.0001 | <0.0001 |  |
| 38 | Left ventral anterior prefrontal cortex | -42.6 | 47.4 | 2.4 | 0.201 | (0.144) | 0.201 | (0.144) | <0.0001 | <0.0001 |  |
| 55 | Right intra-parietal sulcus | 32.4 | -59.4 | 41.4 | 0.255 | (0.188) | 0.255 | (0.188) | <0.0001 | <0.0001 |  |
| 70 | Left thalamus | -12 | -3 | 12.75 | 0.199 | (0.157) | 0.199 | (0.157) | <0.0001 | <0.0001 |  |
| 87 | Left temporoparietal junction | -51.75 | -63 | 15 | 0.435 | (0.240) | 0.435 | (0.240) | <0.0001 | <0.0001 |  |
| Nodal strength comparison between the RP-R and HC at the maximum threshold t=4.2  **Note:** Nodal strength was calculated by absolute values of FC; FC, Functional connectivity; HC, Healthy controls; p-unc, uncorrected p-value; p-bonf, Bonferroni corrected p-value (n= node number of a significant component); RP-R, Recovered patients with relapsed; sd, standard deviation; | | | | | | | | | | |  |
|  |  |  |  |  |  |  |  |  |  |  |  |
|  |  |  |  |  |  |  |  |  |  |  |  |
|  |  |  |  |  |  |  |  |  |  |  |  |

| **Table S19a. Comparison of altered connected component between the RP-M (n=13) and RP-R (n=12).** | | | | | | | |  |
| --- | --- | --- | --- | --- | --- | --- | --- | --- |
| **Node index** | | **Node label** | **RP-M** | | **RP-R** | | **t value** |  |
| **"i "** | **" j"** | **"i to j"** | **mean(±sd)** | | **mean(±sd)** | |  |
| **Connected component 1 (p=0.0018)** | | | **0.064** | **(0.095)** | **0.369** | **(0.093)** |  |  |
| 15 | 37 | Right fusiform to Right ventral anterior prefrontal cortex. | -0.055 | (0.176) | 0.264 | (0.167) | 3.95 |  |
| 10 | 76 | Right superior frontal cortex to Left posterior insula. | 0.014 | (0.159) | 0.351 | (0.243) | 4.38 |  |
| 15 | 76 | Right fusiform to Left posterior insula. | 0.162 | (0.207) | 0.412 | (0.203) | 2.98 |  |
| 46 | 143 | Right dorsal prefrontal cortex to Left lateral cerebellum | -0.015 | (0.243) | 0.283 | (0.178) | 3.98 |  |
| 50 | 143 | Left posterior parietal to Left lateral cerebellum. | 0.118 | (0.241) | 0.427 | (0.260) | 2.84 |  |
| 54 | 143 | Left intra-parietal sulcus to Left lateral cerebellum. | -0.018 | (0.154) | 0.295 | (0.145) | 4.62 |  |
| 55 | 143 | Right intra-parietal sulcus to Left lateral cerebellum. | 0.018 | (0.190) | 0.335 | (0.261) | 3.39 |  |
| 15 | 144 | Right fusiform to Left lateral cerebellum. | 0.284 | (0.222) | 0.556 | (0.209) | 2.66 |  |
| 46 | 144 | Right dorsal prefrontal cortex to Left lateral cerebellum. | 0.019 | (0.167) | 0.251 | (0.227) | 3.17 |  |
| 15 | 150 | Right fusiform to Left medial cerebellum. | 0.132 | (0.252) | 0.493 | (0.216) | 3.74 |  |
| 44 | 150 | Left dorsolateral prefrontal cortex to Left medial cerebellum. | 0.045 | (0.186) | 0.391 | (0.192) | 3.91 |  |
| Post hoc test between connectomes of the RP-M and RP-M, (p<0.05/3 and 2.0≤t≤3.0) with 10000 permutations and covariates (age and sex)  at the median threshold t=2.5, RP-R had significantly higher FC of a connected component than HC.  **Note:** FC, Functional connectivity; RP-M, Recovery patients with maintained; RP-R, Recovered patients with relapsed; sd, standard deviation; | | | | | | | |  |
|  |
|  |
|  |

| **Table S19b. Nodal strength in significantly different connected components between the RP-M (n=13) and RP-R (n=12).** | | | | | | | | | | |  |
| --- | --- | --- | --- | --- | --- | --- | --- | --- | --- | --- | --- |
| **Node index** | **Node label** | **Coordinate** | | | **RP-M** | | **RP-R** | | **p-unc** | **p-bonf** |  |
|  |  |  |  |  | **mean(±sd)** | | **mean(±sd)** | |  |  |  |
| 10 | Right superior frontal cortex | 23.0625 | 33 | 47.0625 | 0.130 | (0.086) | 0.350 | (0.244) | 0.0056 | 0.0678 |  |
| 15 | Left ventral medial prefrontal cortex | 27.9375 | -36.9375 | -15 | 0.874 | (0.387) | 1.754 | (0.569) | 0.0001 | 0.0017 |  |
| 37 | Right ventral anterior prefrontal cortex | 42 | 48 | -3 | 0.139 | (0.114) | 0.294 | (0.102) | 0.0017 | 0.0200 |  |
| 44 | Left dorsolateral prefrontal cortex | -44.25 | 27 | 33 | 0.157 | (0.101) | 0.392 | (0.192) | 0.0008 | 0.0092 |  |
| 46 | Right dorsal prefrontal cortex | 44.4 | 8.4 | 33.6 | 0.332 | (0.204) | 0.571 | (0.319) | 0.0346 | 0.4155 |  |
| 50 | Left posterior parietal | -35.0625 | -45.9375 | 48 | 0.212 | (0.156) | 0.427 | (0.261) | 0.0193 | 0.2317 |  |
| 54 | Left intra-parietal sulcus | -32.4 | -57.6 | 45.6 | 0.126 | (0.084) | 0.296 | (0.146) | 0.0014 | 0.0174 |  |
| 55 | Right intra-parietal sulcus | 32.4 | -59.4 | 41.4 | 0.145 | (0.117) | 0.334 | (0.263) | 0.0270 | 0.3243 |  |
| 76 | Left posterior insula | -30 | -27.75 | 9 | 0.339 | (0.143) | 0.761 | (0.403) | 0.0017 | 0.0203 |  |
| 143 | Left lateral cerebellum | -27.6 | -44.4 | -24.6 | 0.686 | (0.270) | 1.341 | (0.667) | 0.0034 | 0.0408 |  |
| 144 | Left lateral cerebellum | -24 | -54 | -21 | 0.439 | (0.234) | 0.843 | (0.296) | 0.0009 | 0.0110 |  |
| 150 | Left medial cerebellum | -15.9375 | -63.9375 | -21 | 0.374 | (0.173) | 0.885 | (0.342) | 0.0001 | 0.0010 |  |
| Nodal strength comparison between the RP-M and RP-R at the median threshold t=2.5  **Note:** Nodal strength was calculated by absolute values of FC; FC, Functional connectivity; RP-M, Recovery patients with maintained; RP-R, Recovered patients with relapsed; p-unc, uncorrected p-value; p-bonf, Bonferroni corrected p-value (n= node number of a significant component); sd, standard deviation; | | | | | | | | | | |  |
|  |  |  |  |  |  |  |  |  |  |  |  |
|  |  |  |  |  |  |  |  |  |  |  |  |
|  |  |  |  |  |  |  |  |  |  |  |  |

| **Table S20a. Comparison of altered connected component between the RP-M (n=13) and RP-R (n=12).** | | | | | | | |  |
| --- | --- | --- | --- | --- | --- | --- | --- | --- |
| **Node index** | | **Node label** | **RP-M** | | **RP-R** | | **t value** |  |
| **"i "** | **" j"** | **"i to j"** | **mean(±sd)** | | **mean(±sd)** | |  |
| **Connected component 1 (p=0.012)** | | | **0.041** | **(0.076)** | **0.383** | **(0.094)** |  |  |
| 15 | 37 | Right fusiform to Right ventral anterior prefrontal cortex. | -0.055 | (0.176) | 0.264 | (0.167) | 3.95 |  |
| 15 | 150 | Right fusiform to Left medial cerebellum. | 0.132 | (0.252) | 0.493 | (0.216) | 3.74 |  |
| 44 | 150 | Left dorsolateral prefrontal cortex to Left medial cerebellum. | 0.045 | (0.186) | 0.391 | (0.192) | 3.91 |  |
| **Connected component 2 (p=0.006)** | | | **0.001** | **(0.018)** | **0.291** | **(0.030)** |  |  |
| 46 | 143 | Right dorsal prefrontal cortex to Left lateral cerebellum | -0.015 | (0.243) | 0.283 | (0.178) | 3.98 |  |
| 54 | 143 | Left intra-parietal sulcus to Left lateral cerebellum. | -0.018 | (0.154) | 0.295 | (0.145) | 4.62 |  |
| 55 | 143 | Right intra-parietal sulcus to Left lateral cerebellum. | 0.018 | (0.190) | 0.335 | (0.261) | 3.39 |  |
| 46 | 144 | Right dorsal prefrontal cortex to Left lateral cerebellum. | 0.019 | (0.167) | 0.251 | (0.227) | 3.17 |  |
| Posthoc test between connectomes of the RP-M and RP-M, (p<0.05/3 and 2.0≤t≤3.0) with 10000 permutations and covariates (age and sex)  at the maximum threshold t=3.0, RP-R had significantly higher FC of a connected component than HC.  **Note:** FC, Functional connectivity; RP-M, Recovery patients with maintained; RP-R, Recovered patients with relapsed; | | | | | | | |  |
|  |
|  |
|  |

| **Table S20b. Nodal strength in significantly different connected components between the RP-M (n=13) and RP-R (n=12).** | | | | | | | | | | |  |
| --- | --- | --- | --- | --- | --- | --- | --- | --- | --- | --- | --- |
| **Node index** | **Node label** | **Coordinate** | | | **RP-M** | | **RP-R** | | **p-unc** | **p-bonf** |  |
|  |  |  |  |  | **mean(±sd)** | | **mean(±sd)** | |  |  |  |
| 15 | Left ventral medial prefrontal cortex | 27.9375 | -36.9375 | -15 | 0.356 | (0.191) | 0.786 | (0.259) | 0.0001 | 0.0003 |  |
| 37 | Right ventral anterior prefrontal cortex | 42 | 48 | -3 | 0.139 | (0.114) | 0.294 | (0.102) | 0.0017 | 0.0067 |  |
| 44 | Left dorsolateral prefrontal cortex | -44.25 | 27 | 33 | 0.157 | (0.101) | 0.392 | (0.192) | 0.0008 | 0.0031 |  |
| 46 | Right dorsal prefrontal cortex | 44.4 | 8.4 | 33.6 | 0.332 | (0.204) | 0.571 | (0.319) | 0.0346 | 0.1731 |  |
| 54 | Left intra-parietal sulcus | -32.4 | -57.6 | 45.6 | 0.126 | (0.084) | 0.296 | (0.146) | 0.0014 | 0.0072 |  |
| 55 | Right intra-parietal sulcus | 32.4 | -59.4 | 41.4 | 0.145 | (0.117) | 0.334 | (0.263) | 0.0270 | 0.1351 |  |
| 143 | Left lateral cerebellum | -27.6 | -44.4 | -24.6 | 0.474 | (0.162) | 0.914 | (0.432) | 0.0023 | 0.0114 |  |
| 144 | Left lateral cerebellum | -24 | -54 | -21 | 0.129 | (0.102) | 0.287 | (0.172) | 0.0098 | 0.0489 |  |
| 150 | Left medial cerebellum | -15.9375 | -63.9375 | -21 | 0.374 | (0.173) | 0.885 | (0.342) | 0.0001 | 0.0003 |  |
| Nodal strength comparison between RP-M and RP-R at the maximum threshold t=3.0  **Note:** Nodal strength was calculated by absolute values of FC; FC, Functional connectivity; RP-M, Recovery patients with maintained; RP-R, Recovered patients with relapsed; p-unc, uncorrected p-value; p-bonf, Bonferroni corrected p-value (n= node number of a significant component); sd, standard deviation; | | | | | | | | | | |  |
|  |  |  |  |  |  |  |  |  |  |  |  |
|  |  |  |  |  |  |  |  |  |  |  |  |
|  |  |  |  |  |  |  |  |  |  |  |  |

| **Table S21. Spearman correlation between the degree centrality of hubs and PANSS.** | | | | | |  |
| --- | --- | --- | --- | --- | --- | --- |
| **Node index** | **Label of Hubs** | **PANSS** | **rho** | **p-unc** | **p-bonf** |  |
|  |  |  |  |  |  |  |
| **RP** |  |  |  |  |  |  |
|  | FPN | Positive | -0.464 | 0.010 | 0.060 |  |
| 41 | Left anterior cingulate cortex | Positive | -0.464 | 0.010 | 0.220 |  |
| 30 | Left intra-parietal sulcus | Negative | 0.496 | 0.005 | 0.110 |  |
| 135 | Left posterior occipital | Negative | 0.395 | 0.031 | 0.341 |  |
| **RP-M** |  |  |  |  |  |  |
| 58 | Left anterior cingulate cortex | General | 0.589 | 0.034 | 0.918 |  |
|  |  | Total | 0.613 | 0.026 | 0.702 |  |
| 70 | Left thalamus | Negative | 0.577 | 0.039 | 1.000 |  |
| 126 | Right occipital | Negative | -0.577 | 0.039 | 1.000 |  |
| 131 | Left occipital | Negative | 0.630 | 0.021 | 0.567 |  |
| **RP-R** |  |  |  |  |  |  |
| 129 | Right occipital | General | -0.620 | 0.031 | 0.806 |  |
| **Note:** General, General total PANSS; Negative, negative total PANSS; p-unc, uncorrected p-value; p-bonf, Bonferroni corrected p-value; PANSS, Positive and Negative Syndrome Score; Posisive, positive total PANSS; rho, Spearman correlation coefficient; RP, Recovered patients; RP-M, Recovered patients with maintained, RP-R, Recovered patients with relapsed; Total, total PANSS; | | | | | |  |
|  |  |  |  |  |  |  |
|  |  |  |  |  |  |  |

| **Table S22. Spearman correlation between the degree centrality of hubs and cognitive function score.** | | | | | |  |
| --- | --- | --- | --- | --- | --- | --- |
| **Node index** | **Label of hubs** | **CNT** | **rho** | **p-unc** | **p-bonf** |  |
| **RP** |  |  |  |  |  |  |
|  | FPN | Executive function | -0.457 | 0.011 | 0.060 |  |
| 28 | Right angular gyrus cortex | Attention | -0.459 | 0.011 | 0.242 |  |
|  |  | Executive function | -0.411 | 0.024 | 0.528 |  |
| 41 | Left anterior cingulate cortex | Executive function | -0.457 | 0.011 | 0.242 |  |
| 93 | Supplementary motor area | Language | -0.424 | 0.020 | 0.440 |  |
| **RP-M** |  |  |  |  |  |  |
|  | Mean degree centrality of hubs | Executive function | -0.606 | 0.028 | 0.028 |  |
|  | DMN | Executive function | -0.581 | 0.037 | 0.222 |  |
|  | FPN | Verbal memory | -0.555 | 0.049 |  |  |
|  |  | Executive function | -0.846 | 0.000 | <0.0001 |  |
|  |  | Global | -0.714 | 0.006 |  |  |
|  | CON | Executive function | -0.584 | 0.036 | 0.216 |  |
| 3 | Left anterior prefrontal cortex | Executive function | -0.670 | 0.012 | 0.324 |  |
| 16 | Left precuneus | Executive function | -0.840 | 0.000 | <0.0001 |  |
|  |  | Global | -0.665 | 0.013 |  |  |
| 41 | Left anterior cingulate cortex | Verbal memory | -0.555 | 0.049 | 1.000 |  |
|  |  | Executive function | -0.846 | 0.000 | <0.0001 |  |
|  |  | Global | -0.714 | 0.006 | 0.162 |  |
| 58 | Left anterior cingulate cortex | Language | -0.808 | 0.011 | 0.067 |  |
| 63 | Left basal ganglia | Executive function | -0.598 | 0.031 | 0.837 |  |
| 64 | Left intra-parietal sulcus | Attention | 0.610 | 0.027 | 0.729 |  |
|  |  | Executive function | 0.798 | 0.001 | 0.027 |  |
|  |  | Global | 0.736 | 0.004 | 0.108 |  |
| 72 | Right thalamus | Executive function | -0.570 | 0.042 | 1.000 |  |
| 111 | Left temporal | Attention | -0.659 | 0.014 | 0.378 |  |
| 142 | Left posterior occipital | Verbal memory | -0.632 | 0.021 | 0.567 |  |
| 154 | Left med-cerebellum | Verbal memory | -0.577 | 0.039 | 1.000 |  |
| **RP-R** |  |  |  |  |  |  |
| 28 | Right angular gyrus cortex | Attention | 0.615 | 0.033 | 0.891 |  |
| 72 | Right thalamus | Attention | -0.580 | 0.048 | 1.000 |  |
| 75 | Right basal ganglia | Executive function | 0.624 | 0.030 | 0.810 |  |
|  |  | Language | 0.720 | 0.008 | 0.216 |  |
| **Note:** CNT, Computerized Neurocognitive test; rho, Spearman correlation coefficient; p-unc, uncorrected p-value; p-bonf, Bonferroni corrected p-value; RP, Recovered patients; RP-M, Recovered patients with maintained, RP-R, Recovered patients with relapsed. | | | | | |  |
|  |  |  |  |  |  |  |
|  |  |  |  |  |  |  |

| **Table S23. Spearman correlation between the connectivity strength and PANSS.** | | | | | | |  |
| --- | --- | --- | --- | --- | --- | --- | --- |
| **Node index** | | **Node label** | **PANSS** | **rho** | **p-unc** | **p-bonf** |  |
| **"i "** | **" j"** | **"i to j"** |  |  |  |  |  |
| **RP** |  |  |  |  |  |  |  |
| 65 | 66 | Left ventral frontal cortex to Left basal ganglia | General | 0.542 | 0.002 | 0.010 |  |
|  |  |  | Total | 0.537 | 0.002 | 0.010 |  |
| 70 | 131 | Left thalamus to Left occipital | Negative | 0.428 | 0.018 | 0.090 |  |
| **RP-R** |  |  |  |  |  |  |  |
| 14 | 32 | Right anterior-posterior cingulate cortex to Left superior frontal cortex** | Positive | -0.673 | 0.016 | 0.400 |  |
| 46 | 143 | Right dorsal prefrontal cortex to Left lateral cerebellum* | Positive | 0.732 | 0.007 | 0.077 |  |
| **Significantly altered connectivity between the RP-R and HC. *Significantly altered connectivity between the RP-M and RP-R.  **Note:** General, General total PANSS; Negative, negative total PANSS; p-unc, uncorrected p-value; p-bonf, Bonferroni corrected p-value; PANSS, Positive and Negative Syndrome Score; Positive, positive total PANSS; rho, Spearman correlation coefficient; RP, Recovered patients; RP-M, Recovered patients with maintained, RP-R, Recovered patients with relapsed; Total, total PANSS; | | | | | | |  |
|  |  |  |  |  |  |  |  |
|  |  |  |  |  |  |  |  |

| **Table S24. Spearman correlation between the connectivity strength and cognitive function score.** | | | | | | |  |
| --- | --- | --- | --- | --- | --- | --- | --- |
| **Node index** | | **Node label** | **CNT** | **rho** | **p-unc** | **p-bonf** |  |
| **"i "** | **" j"** | **"i to j"** |  |  |  |  |  |
| **RP-M** | |  |  |  |  |  |  |
| 55 | 143 | Right intra-parietal sulcus to Left lateral cerebellum* | Attention | 0.564 | 0.045 | 0.495 |  |
| 15 | 150 | Right fusiform to Left medial cerebellum* | Verbal memory | -0.624 | 0.023 | 0.259 |  |
| 44 | 150 | Left dorsolateral prefrontal cortex to Left medial cerebellum* | Verbal memory | 0.599 | 0.031 | 0.341 |  |
| **RP-R** | |  |  |  |  |  |  |
| 33 | 38 | Left occipital to Left ventral anterior prefrontal cortex** | Global | -0.601 | 0.039 | 0.975 |  |
| 14 | 87 | Right posterior cingulate cortex to left temporoparietal junction** | Verbal memory | -0.785 | 0.003 | 0.075 |  |
|  |  |  | Attention | -0.630 | 0.028 | 0.700 |  |
|  |  |  | Global | -0.683 | 0.014 | 0.350 |  |
| 46 | 143 | Right dorsal prefrontal cortex to Left lateral cerebellum* | Attention | 0.748 | 0.005 | 0.055 |  |
|  |  |  | Executive function | 0.751 | 0.005 | 0.055 |  |
|  |  |  | Language | 0.720 | 0.008 | 0.088 |  |
|  |  |  | Global | 0.818 | 0.001 | 0.011 |  |
| **Significantly altered connectivity between the RP-R and HC. *Significantly altered connectivity between the RP-M and RP-R.  **Note** CNT, Computerized Neurocognitive test; rho, Spearman correlation coefficient; p-unc, uncorrected p-value; p-bonf, Bonferroni corrected p-value; RP, Recovered patients; RP-M, Recovered patients with maintained, RP-R, Recovered patients with relapsed. | | | | | | |  |
|  |  |  |  |  |  |  |  |
|  |  |  |  |  |  |  |  |

| **Table S25. Comparison of subgroup analyses with and without medication free period as an additional covariate** | | | | | | | | | | |  |
| --- | --- | --- | --- | --- | --- | --- | --- | --- | --- | --- | --- |
| **Metrics** |  | **RP-M vs RP-R vs HC** | | **RP-M vs HC** | | **RP-R vs HC** | | **RP-M vs RP-R** | | **Note** |  |
|  |  | p*-value | p-value | p*-value | p-value | p*-value | p-value | p*-value | p-value |  |  |
| **Global** | |  |  |  |  |  |  |  |  |  |  |
| Global efficiency | | 0.0248 | 0.025 | 1.0000 | 1.0000 | 0.0098 | 0.0160 | 0.4462 | 0.3730 | Fig. S2 |  |
| Local efficiency | | 0.3295 | 0.344 |  |  |  |  |  |  | Fig. S3 |  |
| Clustering coefficient | | 0.0620 | 0.066 |  |  |  |  |  |  | Fig. S4 |  |
| Normalized clustering coefficient | | 0.4242 | 0.385 |  |  |  |  |  |  | Fig. S5 |  |
| Characteristic pathlength | | 0.0276 | 0.027 | 1.0000 | 1.0000 | 0.0098 | 0.0110 | 0.5060 | 0.4000 | Fig. S6 |  |
| Normalized characteristic pathlength | | 0.2528 | 0.270 |  |  |  |  |  |  | Fig. S7 |  |
| Small-worldness | | 0.3299 | 0.302 |  |  |  |  |  |  | Fig. S8 |  |
| **Local** | |  |  |  |  |  |  |  |  |  |  |
| Degree centrality | | no significant | |  |  |  |  |  |  | after applied FDR correction, Table S6 |  |
| Betweenness centrality | Left precuneus | 0.0192 | 0.0191 | 1.0000 | 1.0000 | 0.0002 | 0.0003 | 0.1013 | 0.1305 | after applied FDR correction, Table S7 |  |
| Nodal efficiency | | no significant | |  |  |  |  |  |  | after applied FDR correction, Table S8 |  |
| **Network and Functional connectivity** | |  |  |  |  |  |  |  |  |  |  |
| Robustness |  | 0.0090 | 0.0087 | 1.0000 | 1.0000 | 0.0107 | 0.0036 | 0.2167 | 0.2242 | Robustness to targeted attack. Fig. 2 |  |
| Network based Statistics | CC1 | 0.0430 | 0.0360 | 1.0000 | 1.0000 | 0.0360 |  | 0.0020 | 0.0050 | Number of p* and p values are corresponding to number of connected components. Fig. 4 and Table S15-S20 |  |
|  | CC2 |  |  |  |  | <0.001 | <0.001 |  |  |  |  |
|  | CC3 |  |  |  |  | 0.0010 |  |  |  |  |  |
|  | CC4 |  |  |  |  | 0.0010 | 0.0030 |  |  |  |  |
| **Note:** CC, Connected component; HC, Healthy controls; RP-M, Recovered patients with maintained; RP-R, Recovered patients with relapsed; p***-value,** ANOVA (p<0.05) among three groups with covariates of age and sex; p-value, ANOVA (p<0.05) among three groups with covariates of age, sex, and medication free period. | | | | | | | | | | |  |
|  |  |  |  |  |  |  |  |  |  |  |  |
|  |  |  |  |  |  |  |  |  |  |  |  |

**Supplemental figure legends**

**Fig. S1: Flowchart of the procedure.**

**Fig. S2: Summary statistics of global metrics in the main group:** A) Eg, Global efficiency; B) Eloc, Local efficiency; C) Cp, Clustering coefficient; D) Gamma, Normalized clustering coefficient; E) Lp, Characteristic path length; F) Lambda, Normalized characteristic path length; G) Sigma, Small-worldness; and H) Comparison of global metrics (AUCs) between the RP and HC.

**Note:** AUC, area under the curve; HC, Healthy controls; RP, Recovered patients.

**Fig. S3: Summary statistics of global metrics in the subgroups:** A) Eg, Global efficiency; B) Eloc, Local efficiency; C) Cp, Clustering coefficient; D) Gamma, Normalized clustering coefficient; E) Lp, Characteristic path length; F) Lambda, Normalized characteristic path length; G) Sigma, Small-worldness; and H) Comparison of global metrics (AUCs) among the RP-M, RP-R and HC, Eg and Lp were significant (p=0.025, p=0.028) for the ANCOVA (in the post-hoc tests, *p=0.01 for the RP-R vs. HC).

**Note:** ANCOVA, Analysis of covariance; AUC, area under the curve; HC, Healthy controls; RP-M, Recovered patients with maintained; RP-R, Recovered patients with relapsed;

**Fig. S4: A) Degree centrality; B) Betweenness centrality; and C) Nodal efficiency;** Comparison of nodal metrics (AUCs) between the RP and HC using a two-sample t-test (p<0.05) with covariates (age and sex). Significant results were found at the uncorrected level.

**Note:** Colours indicate the direction of significance. AUC, area under the curve; HC, Healthy controls; RP, Recovered patients; vFC, ventral frontal cortex.

**Fig. S5: Degree centrality (AUC of Dc):** Comparison of the Dc among the RP-M, RP-R, and HC A) ANCOVA with covariates (age and sex); and results of the Bonferroni post hoc test between B) the RP-M vs HC; C) RP-R vs. HC; and D) RP-M vs. RP-R. Significant results were found only at the uncorrected level.

**Note:** The colors of the node indicate subnetwork of the brain connectome and direction of significance; ANCOVA, Analysis of covariance; AUC, area under the curve; Cere, Cerebellum network; CON, Cingulo-opercular network; Dc, Degree centrality; DMN, Default mode network; FPN, Frontal parietal network; HC, Healthy controls; Occi, Occipital network; RP, Recovered patients; RP-M, Recovered patients with maintained; RP-R, Recovered patients with relapsed; Sens, Sensorimotor network; vPFC, ventral prefrontal cortex;

**Fig. S6: Betweenness centrality (AUC of Bc):** Comparison of the Bc among the RP-M, RP-R, and HC A) ANCOVA with covariates (age and sex); and results of the Bonferroni post hoc test between B) the RP-M vs. HC; C) RP-R vs. HC; and D) RP-M vs. RP-R. Significant results were found at the uncorrected level except the left precuneus.

**Note:** The colors of the node indicate subnetwork of the brain connectome and direction of significance; ANCOVA, Analysis of covariance; AUC, area under the curve; Bc, Betweenness centrality; Cere, Cerebellum network; CON, Cingulo-opercular network; DMN, Default mode network; FPN, Frontal parietal network; HC, Healthy controls; Occi, Occipital network; RP, Recovered patients; RP-M, Recovered patients with maintained; RP-R, Recovered patients with relapsed; Sens, Sensorimotor network;

**Fig. S7: Nodal efficiency (AUC of Ne):** Comparison of the Ne among the RP-M, RP-R, and HC A) ANCOVA with covariates (age and sex); and results of the Bonferroni post hoc test between B) RP-M vs. HC; C) RP-R vs. HC; and D) RP-M vs. RP-R. Significant results were found only at the uncorrected level.

**Note:** The colors of the node indicate subnetwork of the brain connectome and direction of significance; ANCOVA, Analysis of covariance; AUC, area under the curve; Cere, Cerebellum network; CON, Cingulo-opercular network; DMN, Default mode network; FPN, Frontal parietal network; HC, Healthy controls; Occi, Occipital network; RP, Recovered patients; RP-M, Recovered patients with maintained; RP-R, Recovered patients with relapsed; Sens, Sensorimotor network; vFC, ventral frontal cortex; vPFC, ventral prefrontal cortex;

**Fig. S8: Robustness to random failure:** A) in the RP; B) in the RP-M; C) in the RP-R; and D) in the HC; At the range thresholds, the changes of GCC size were no different.

**Note**: GCC, giant connected component; HC, Healthy controls; RP-M, Recovered patients with maintained; RP-R, Recovered patients with relapsed;
