# Supplementary figures and images for: Network biomarkers in recovered psychosis patients who discontinued antipsychotics
Source: Mol Psychiatry. 2023 Sep 29;28(9):3717–26. doi: 10.1038/s41380-023-02279-6 (PMC10730417; doi:10.1038/s41380-023-02279-6)

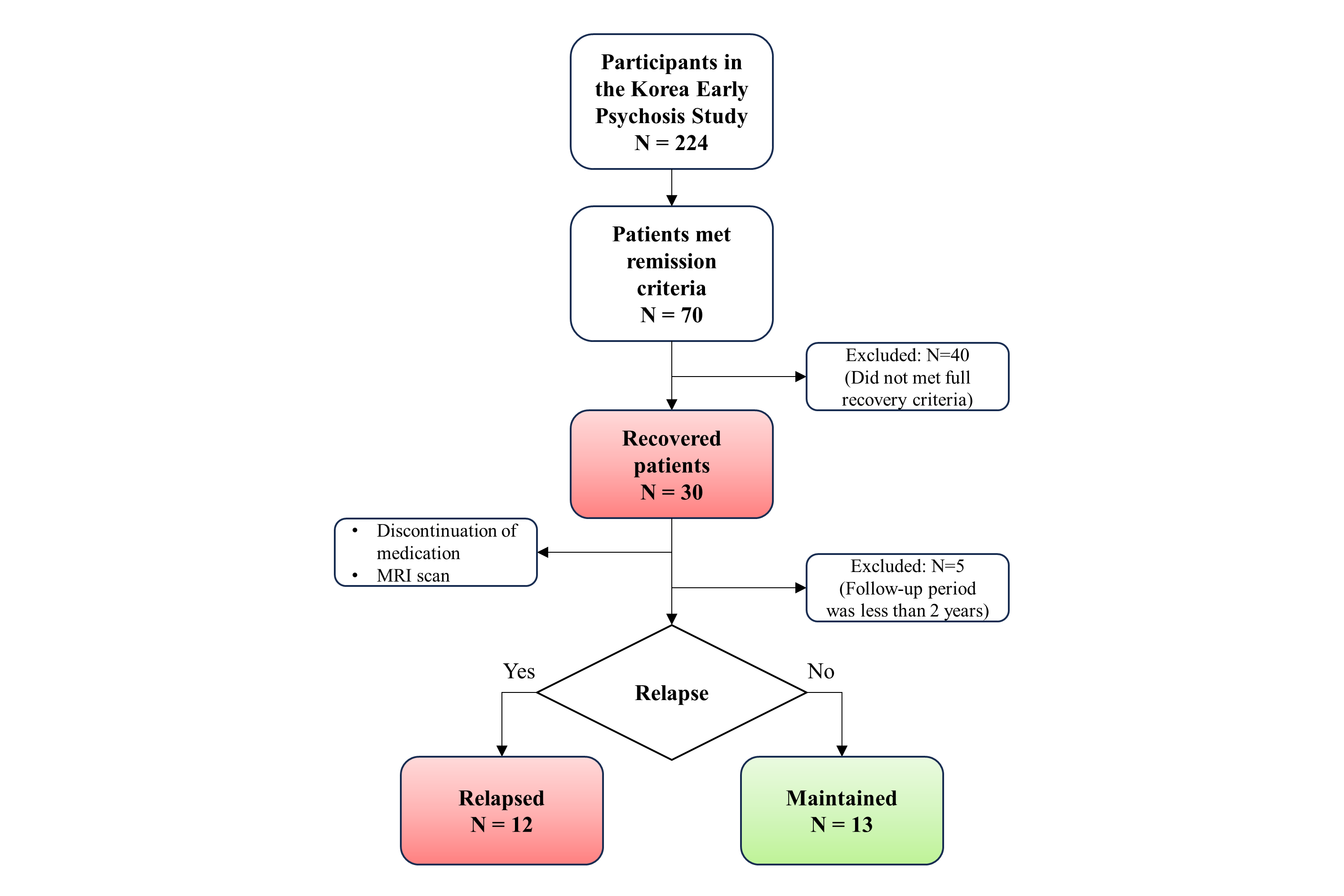

Supplement: Supplementary file 2 — Suppl Figure S1 [file 41380_2023_2279_MOESM2_ESM.tif]

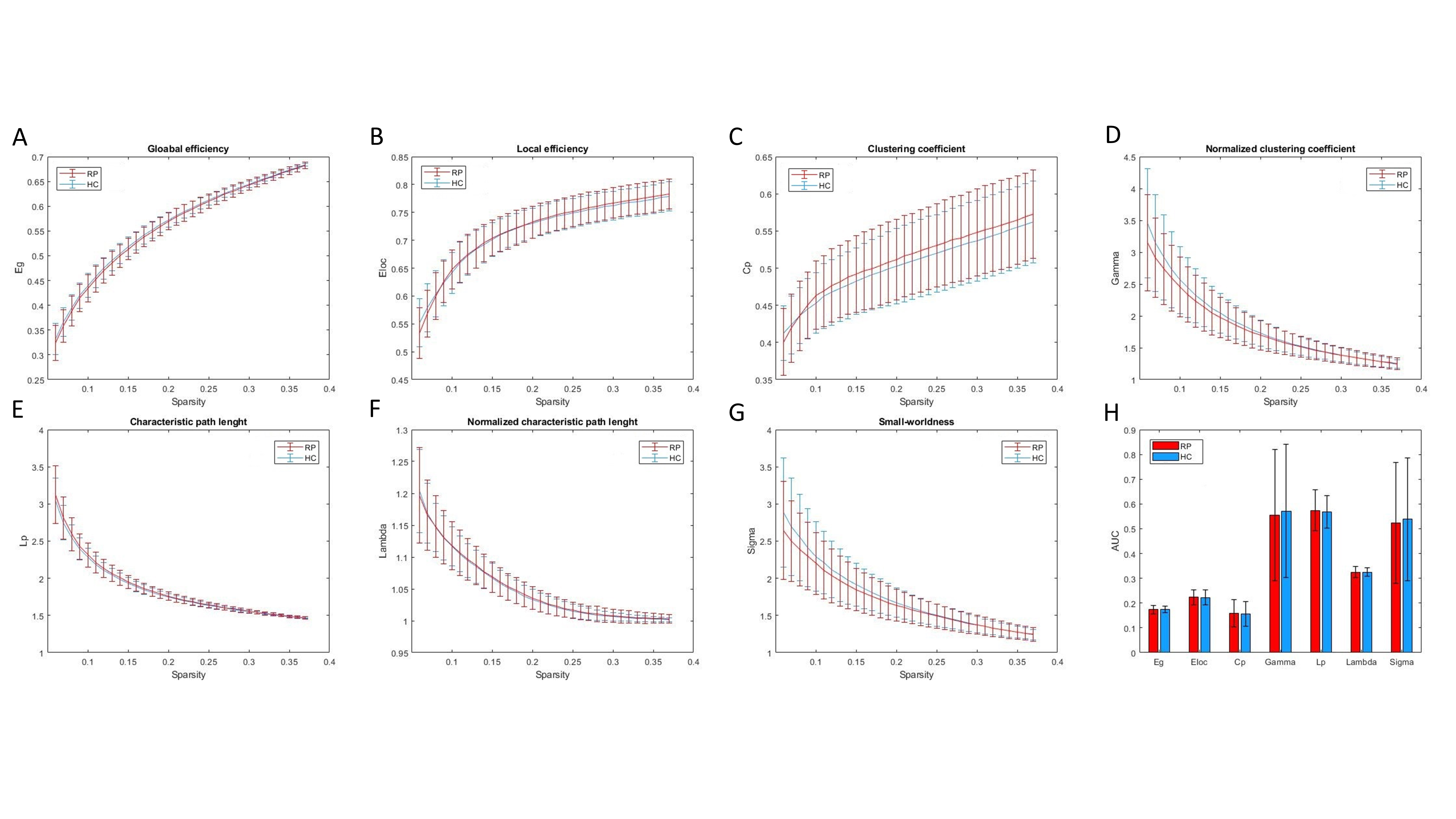

Supplement: Supplementary file 3 — Suppl Figure S2 [file 41380_2023_2279_MOESM3_ESM.tif]

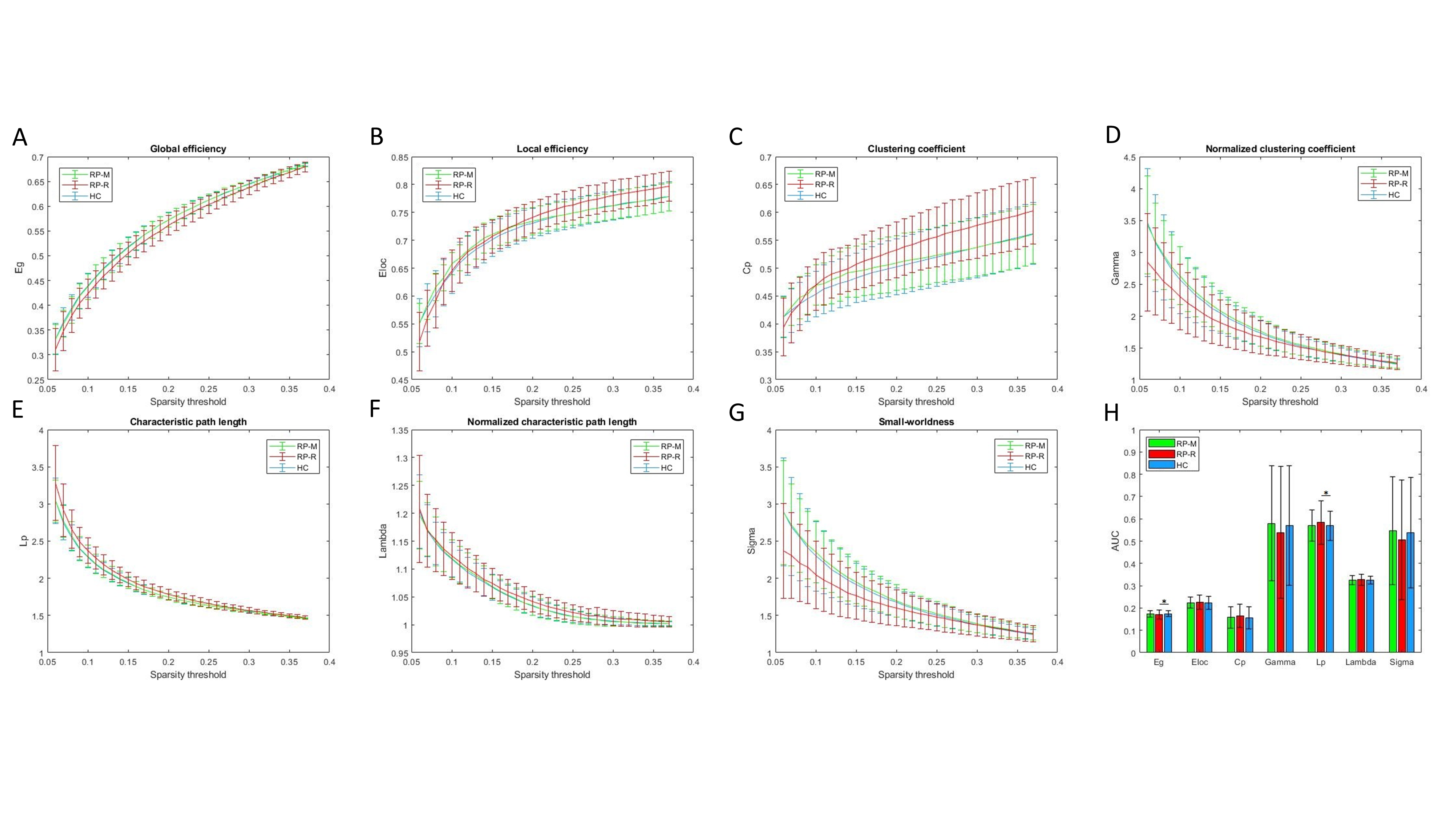

Supplement: Supplementary file 4 — Suppl Figure S3 [file 41380_2023_2279_MOESM4_ESM.tif]

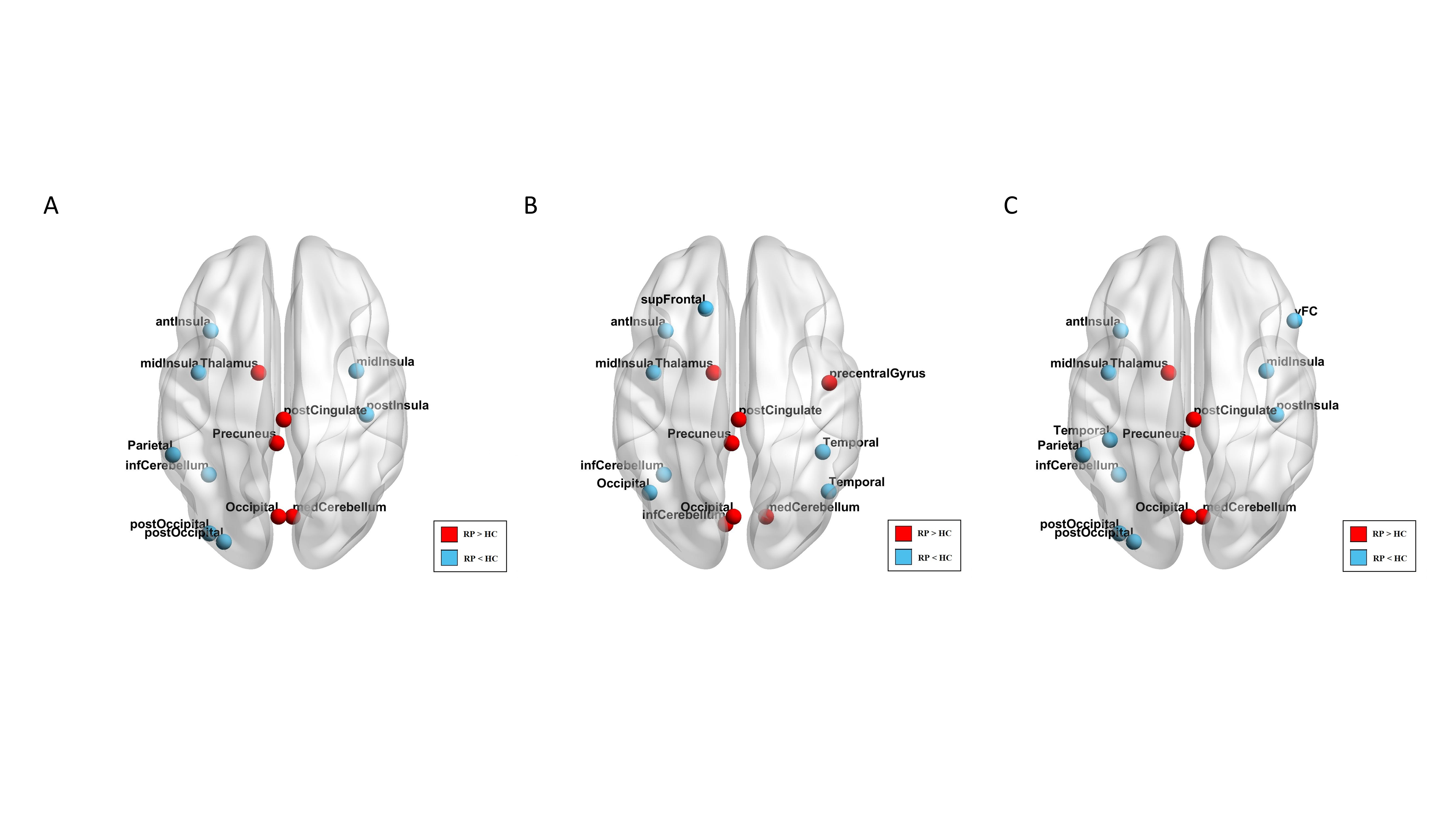

Supplement: Supplementary file 5 — Suppl Figure S4 [file 41380_2023_2279_MOESM5_ESM.tif]

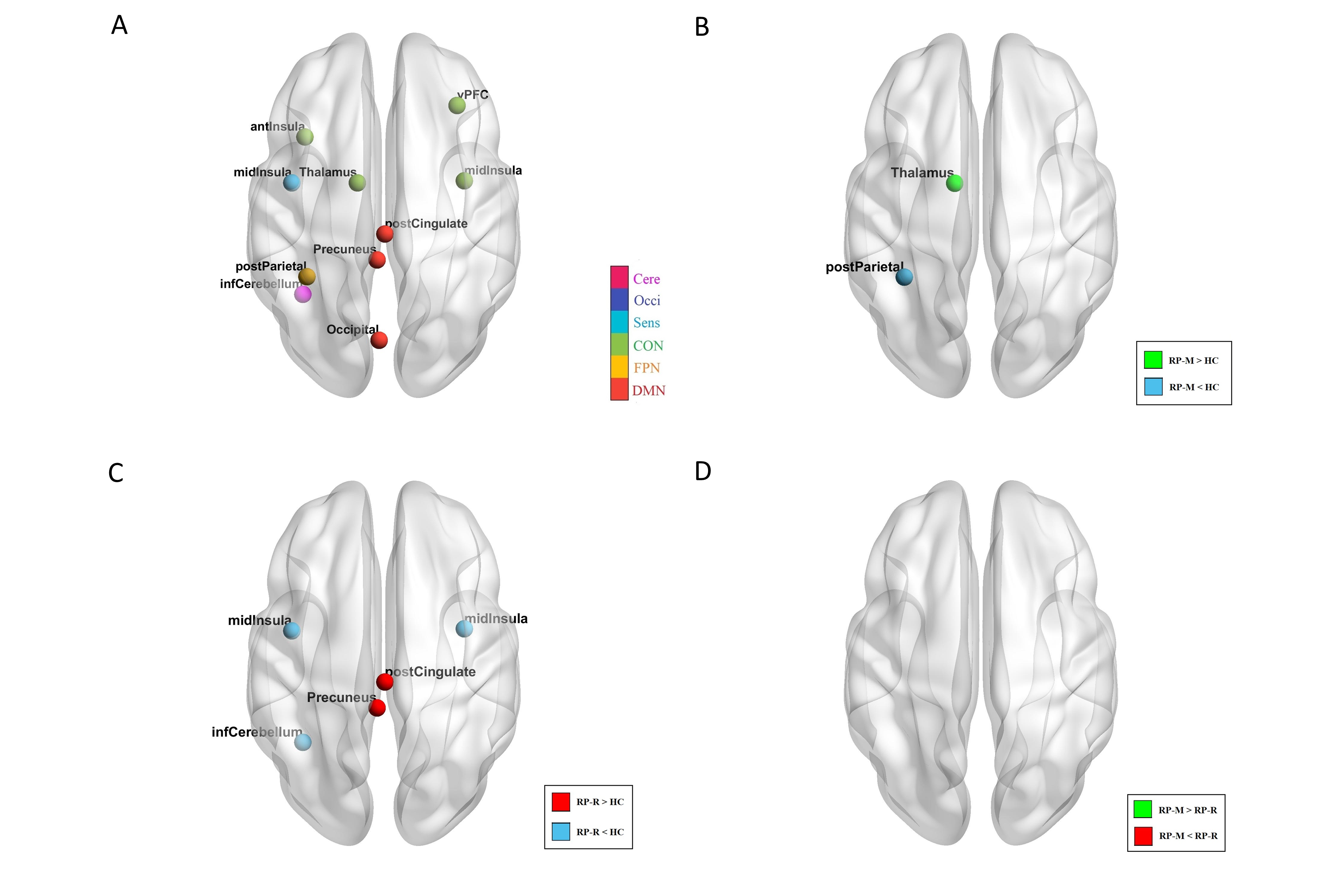

Supplement: Supplementary file 6 — Suppl Figure S5 [file 41380_2023_2279_MOESM6_ESM.tif]

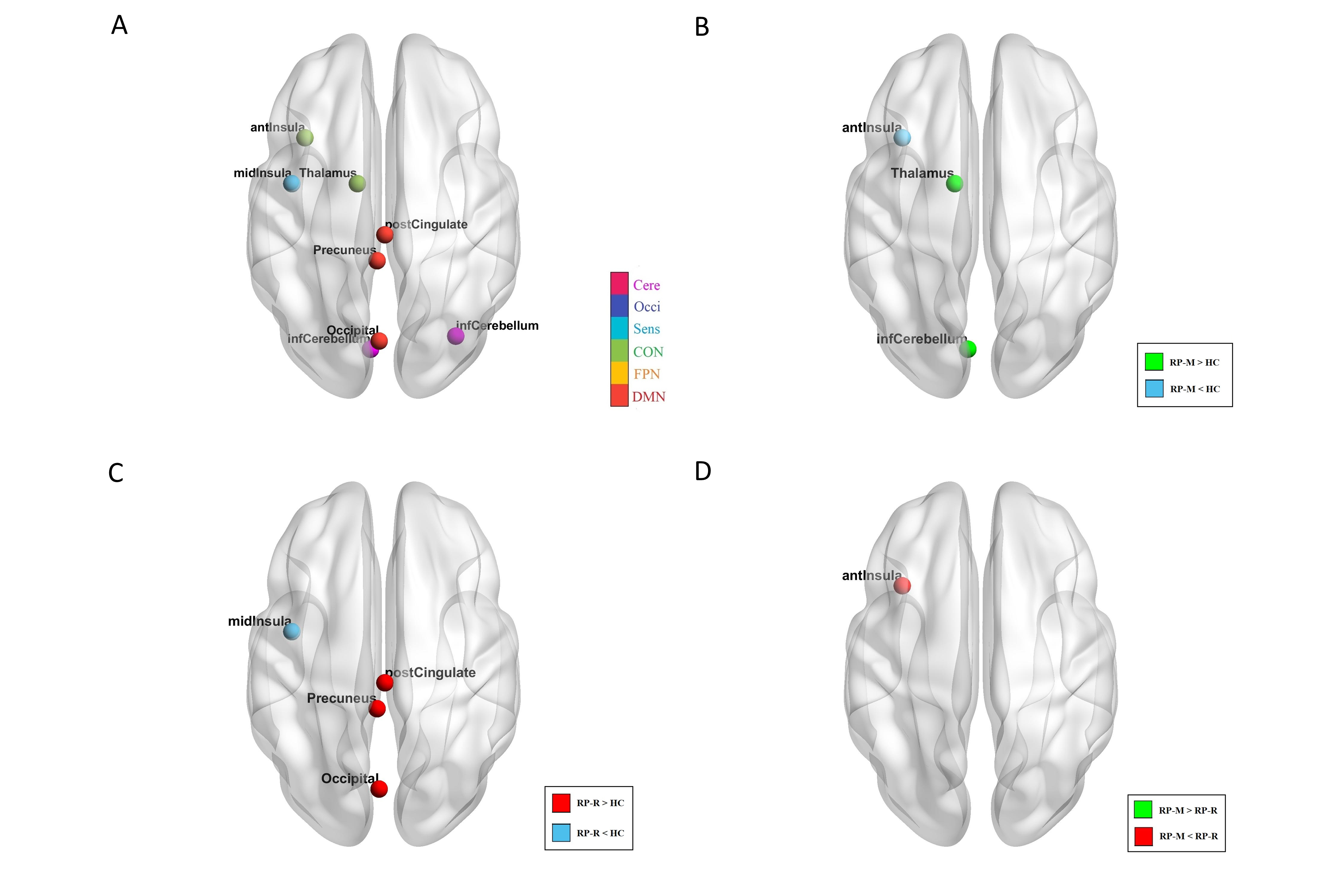

Supplement: Supplementary file 7 — Suppl Figure S6 [file 41380_2023_2279_MOESM7_ESM.tif]

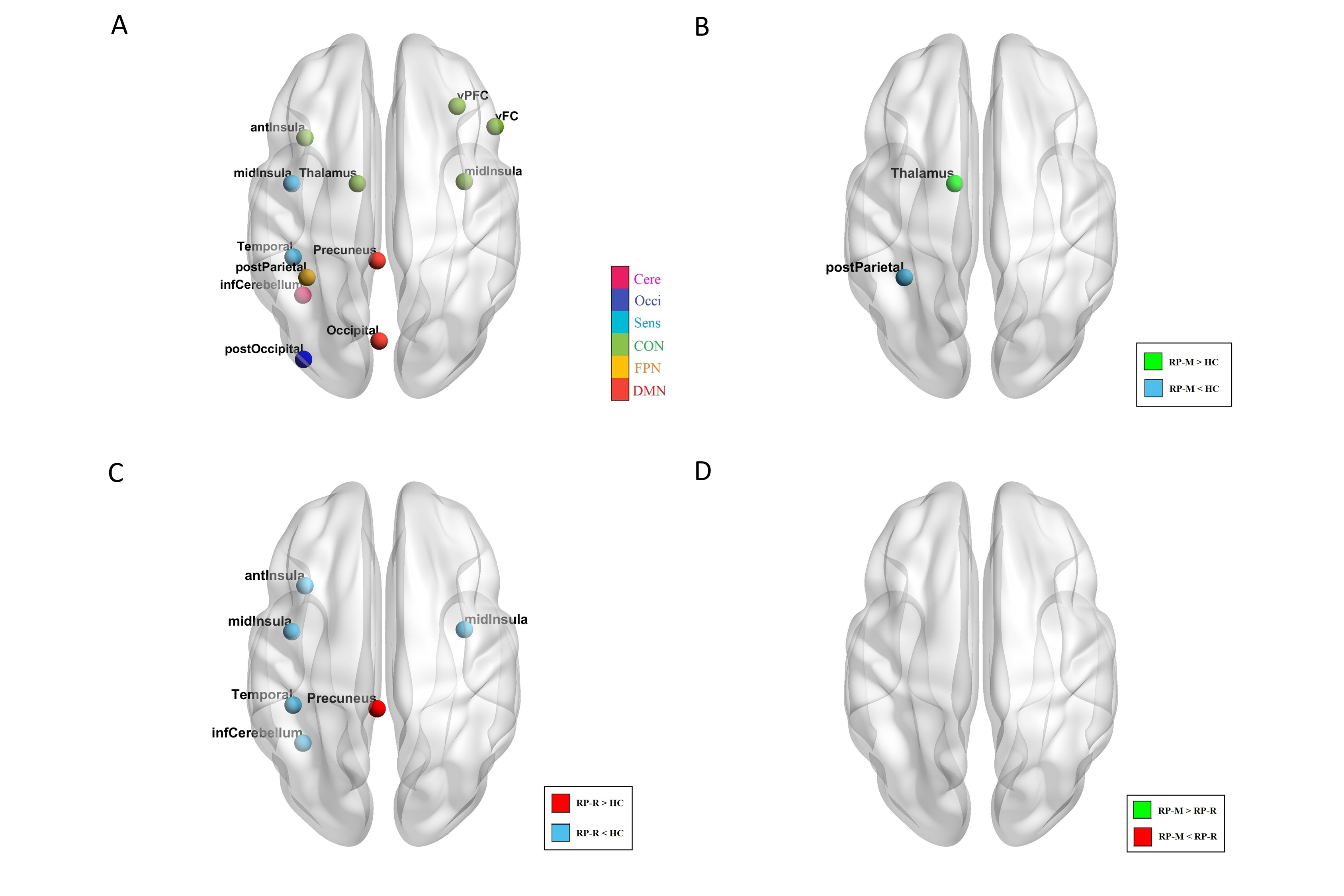

Supplement: Supplementary file 8 — Suppl Figure S7 [file 41380_2023_2279_MOESM8_ESM.tif]

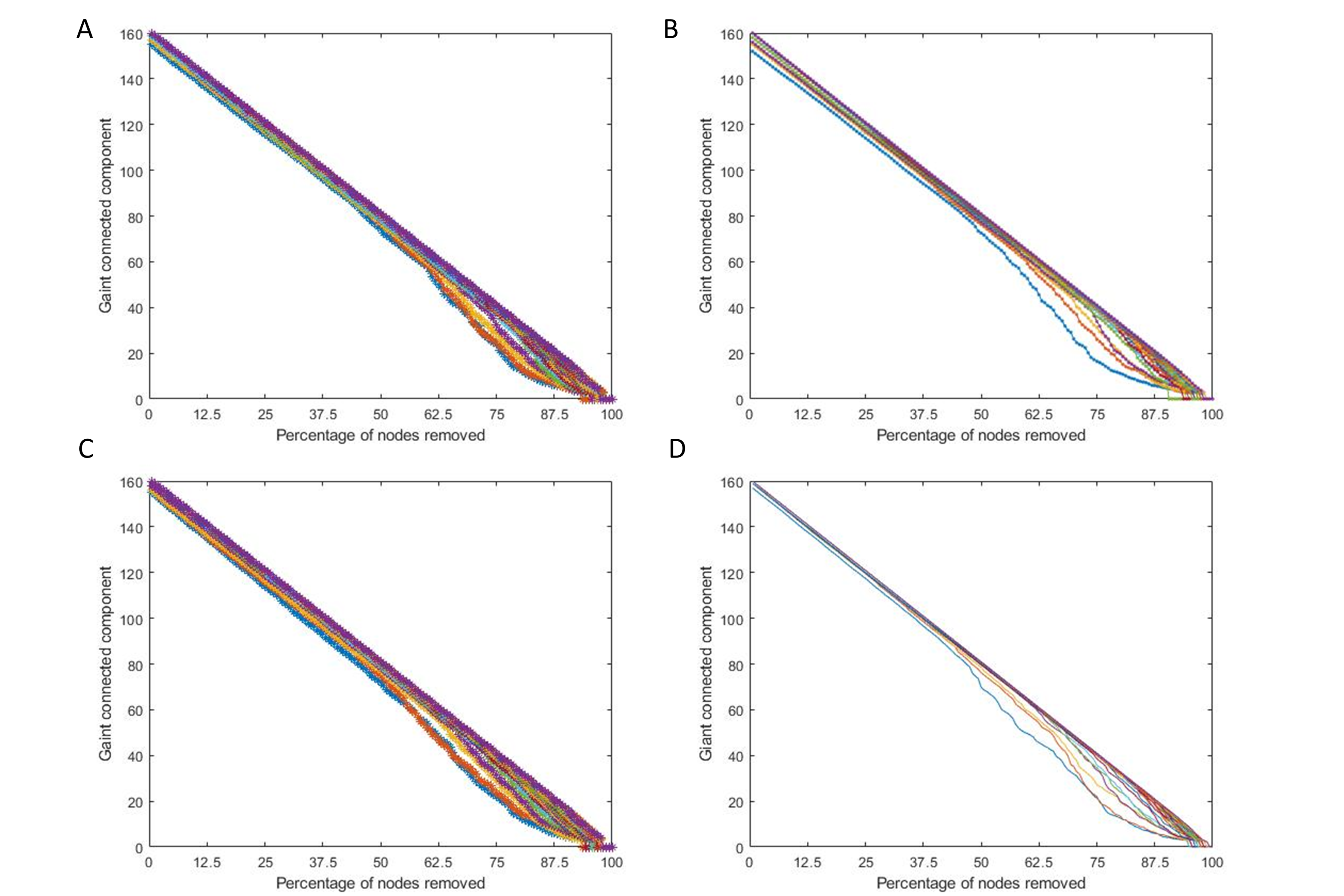

Supplement: Supplementary file 9 — Suppl Figure S8 [file 41380_2023_2279_MOESM9_ESM.tif]
